# Family-based sexual health interventions for adolescents in low- and middle-income countries: systematic analysis and meta-analysis

**DOI:** 10.1093/inthealth/ihaf017

**Published:** 2025-03-21

**Authors:** Dilini Mataraarachchi, Thomas Shepherd, Ram Bajpai, Gayan Ariyadasa, Nadia Corp, Priyamvada Paudyal

**Affiliations:** School of Medicine, Keele University, Keele, Staffordshire ST5 5BG, UK; School of Medicine, Keele University, Keele, Staffordshire ST5 5BG, UK; School of Medicine, Keele University, Keele, Staffordshire ST5 5BG, UK; School of Medicine, Keele University, Keele, Staffordshire ST5 5BG, UK; School of Medicine, Keele University, Keele, Staffordshire ST5 5BG, UK; Institute for Global Health and Wellbeing, School of Medicine, Keele University, Keele, UK

**Keywords:** adolescents, family-based interventions, low- and middle-income countries, sexual and reproductive health

## Abstract

Family-based sexual health interventions (FBSHI) have received considerable attention for their success in promoting adolescent sexual health outcomes. However, their effectiveness in low- and middle-income countries (LMICs) is unclear. Systematic searches were conducted for studies published from January 2000 to October 2023 using five electronic databases. Studies were included if they included adolescents aged 10–19 y and their family members (parents, siblings or primary caregivers) in a key intervention component, evaluated the effectiveness of the interventions using an experimental or quasi-experimental design, assessed sexual and reproductive health outcomes reported by adolescents and were carried out in LMICs. The review included nine studies, with 2404 adolescent participants and their families. Meta-analysis was carried out using a random-effects model. The key themes that emerged from this systematic review were: (i) FBSHI significantly improved adolescents’ sexual health knowledge; and (ii) the impact of FBSHI on molding adolescents’ sexual health attitudes, practices and family communication around sexual health topics was inconsistent. The importance of conducting combined interventions that involved adolescents and their parents to elicit better outcomes was highlighted in the review. Future research should prioritize under-represented geographical regions such as Asia and include culturally adopted, contextually relevant material to increase the acceptability and effectiveness in LMIC settings.

By synthesizing existing literature, this review contributes to gaining a comprehensive understanding of varying strategies that can be used to ensure the effectiveness of family-based sexual health interventions in promoting adolescent sexual health in the LMIC setting. The review also highlights areas not explored by the existing research and that need attention when conducting further research.

## Introduction

Adolescence is a critical development period marked by significant physical, emotional and social changes.^1^ During this time, a child acquires sexual maturation, which makes them vulnerable to sexual risk behaviors.^2^ Initiation of sexual activity, while they lack adequate knowledge and skills on sexual and reproductive health (SRH), puts them at a higher risk of unintended pregnancy, unsafe abortion, sexual harassment and sexually transmitted infections (STIs).^3^ To protect adolescents from the negative consequences of sexual risk behaviors, it is important to equip them with the necessary SRH knowledge and skills.

Despite global efforts to improve adolescent SRH, significant disparities still persist, particularly in low- and middle-income countries (LMICs). While the adolescent fertility rate has declined globally, it has remained stagnant or even increased in some LMICs, particularly in Southeast Asia and Africa, where there is wide between-country variation.^4–6^ According to UNICEF data, the global average for adolescent fertility rate (AFR) among females aged 15–19 y in 2018 is 50 per 1000 girls, with the highest rates in LMICs like Lao PDR (AFR=94), Cambodia (AFR=57), Thailand (AFR=50), and Indonesia (AFR=48).^7^ This has resulted in high maternal and neonatal morbidity and mortality rates in these countries, adding a further burden to healthcare systems and social life. In addition, STIs account for a significant health and economic burden for many LMICs. It is estimated that 75–85% of the global burden of STIs is concentrated in these countries.^8^ The high prevalence of early sexual initiation,^9–11^ lack of comprehensive sexuality education,^12^ as well as poor access to SRH services due to stigma and social taboos, further exacerbate the situation.^13–15^ Moreover, findings from these regions suggest a high risk of sexual victimization among prepubertal and late adolescents, in addition to the reproductive health concerns they face.^16^ Further to the above, with the increased use of smart devices, engagement in cybersex appears to be common among adolescents globally,^17^ and with poor regulatory mechanisms, there is a high prevalence of technology-facilitated sexual violence and abuse in LMICs.^18^ These SRH challenges in LMICs highlight the need for effective SRH interventions that go beyond reproductive health outcomes to address broader aspects of the sexual well-being of adolescents. Attempts to incorporate SRH education into school curricula in many LMICs have often faced challenges due to taboos around the topic, insufficient teaching skills and a lack of human and other resources.^19,20^ Moreover, SRH service provision for adolescents in LMICs presents significant challenges, such as resource constraints, low literacy rates, cultural and societal norms, as well as conservative religious and political influences.^21,22^ In these countries, the role of the family in promoting the SRH of adolescents is becoming increasingly significant. Evidence shows that involving parents and caregivers in the sexual health education of adolescents enables the delivery of personalized messages to adolescents to suit their family and cultural values and individual needs.^23,24^ This will also foster a supportive environment for acquiring sexual health knowledge for adolescents and behavior change. Hence, such initiation may reduce the societal resistance that may arise when providing sexual health information to children.

Family-based sexual health interventions (FBSHI) have proven to be a successful approach to promoting adolescent sexual and reproductive health (ASRH) in high-income countries like the USA and the UK.^24–26^ According to United Nations International technical guidance on sexuality education, comprehensive sexuality education must be age-appropriate, incremental, culturally relevant and context appropriate.^27^ Research shows that initiating sexuality education early and continuing as the child matures is the most effective way of transferring sexual information.^28^ This will avoid the pitfalls of a single intervention during adolescence when the child has already acquired information from other sources. Therefore, empowering family members, particularly parents and caregivers, would be an effective approach to providing age-appropriate sex education for children and adolescents. At the same time, this will promote a facilitating environment for adolescent sexual well-being.

Numerous interventions and programs have been implemented in LMICs to empower families to promote ASRH.^29–31^ These interventions and programs address broader aspects of adolescent sexual well-being, including sexual communication and preventing sexual violence. However, the complex interplay between cultural norms, societal values and family dynamics in LMICs raises questions about the effectiveness of FBSHI in promoting ASRH in such settings.^19,32^ Understanding the effectiveness of such programs in LMICs is critical not only for improving ASRH outcomes, but also for informing policymakers, healthcare workers and educators on the best practices for promoting ASRH.

This systematic review and meta-analysis aims to synthesize and critically evaluate the existing body of literature to explore the effectiveness of FBSHI in improving ASRH outcomes in LMIC settings. The effectiveness of these interventions will be evaluated in terms of their impact on adolescents' sexual health knowledge, attitudes, practices and family sexual communication.

## Methods

The protocol for this systematic review was registered in PROSPERO (registration number: CRD42023474311). We prepared and reported this systematic review in accordance with the Cochrane Collaboration^33^ and the Preferred Reporting Items for Systematic Reviews and Meta-Analyses (PRISMA) guidelines.^34^

### Search strategy

A comprehensive literature search was conducted to identify studies that explored the effectiveness of FBSHI in promoting any SRH outcome among adolescents aged 10–19 y living in LMICs. Relevant databases and search terms were identified using previously published reviews in the field^24,35^ and refined with the help of an information specialist. Electronic searches were performed in MEDLINE, EMBASE, CINAHL, PsycINFO and Web of Science databases to include eligible studies from January 2000 to 25 October 2023. The search strategy was initially developed in MEDLINE, combining search terms related to each of the conceptual categories: adolescent (population), family-based intervention (intervention), sexual and reproductive health (outcome) and low-to-middle-income countries (setting). Database-specific subject headings were used where applicable, in combination with text word searching. The final search strategy was then translated to other databases (Annexure 1). In addition, reference lists of the identified articles^30,31,36–40^ were examined to identify any additional relevant studies. Authors of two identified studies^41,42^ were contacted via E-mail and further information was obtained to assess the eligibility of the studies to include in the review.

### Inclusion and exclusion criteria

Studies were considered eligible for the review if (i) the main focus was on adolescents, aged 10–19 y, as defined by the WHO^1^; (ii) they included family members (parents, siblings or primary caregivers) in a key intervention component; (iii) they evaluated the effectiveness of the interventions using an experimental or quasi-experimental design; (iv) specifically assessed adolescent SRH outcomes reported by adolescents, including improvement in knowledge, attitudes, sexual behavior or sexual communication with caregivers/siblings; (vii) were carried out in a LMIC setting; and (vi) were published in peer-reviewed journals written in English from January 2000 to October 2023. This aligns with the global health focus on ASRH in the LMIC setting, particularly after the introduction of the Millennium Development Goals. The period ensures that the review captures the modern, evidence-based interventions carried out in LMICs during this period. We included studies with varying participant groups, such as female- or male-only adolescent groups and mixed-gender groups, to enhance the comprehensiveness of the review. The diversity in participant groups reflected the real-world application of FBSHI across different demographic contexts. This broad inclusion was necessary to generalize the findings to the LMIC setting.

We excluded studies that reported SRH outcomes as a secondary or minor component of outcome assessment along with other outcomes such as nutrition or addiction. Additionally, studies that relied solely on outcome reports from parents or caregivers, rather than directly from adolescents, were also excluded. Studies that assessed the clinical management aspect of sexual disorders, such as antiretroviral therapy adherence or STI treatment compliance, rather than the preventive aspect or SRH promotion of adolescents, were excluded from the review. Furthermore, studies that solely described the content, feasibility or acceptability of programs were excluded if they did not assess the direct impact of the intervention on ASRH outcomes.

### Study selection

Initially, all retrieved records from each database and those identified from the citation search were imported to Rayyan (Rayyan Systems, Inc., Doha, Quatar),^43^ and duplicates were subsequently removed. Titles and abstracts of all articles were screened independently by two reviewers (DM and GA). Studies that did not fulfill the eligibility criteria were excluded at this stage. Following the independent screening of titles and abstracts, the two reviewers discussed and reached a consensus on studies where there was any disagreement between the two. In cases of disagreement between the reviewers, such articles were retained for further detailed assessment. Additionally, studies in which eligibility could not be determined solely from the abstracts were also carried forward for full-text review.

During the next phase, full articles of the potentially eligible studies were independently evaluated by the same two reviewers (DM and GA). Any disagreement regarding study inclusion during the full-text review was resolved through discussion, involving an additional reviewer (TS) when necessary. This approach ensured a thorough and collaborative selection process to determine the final set of studies included in the analysis.

### Data collection

Key information was extracted from each study and reported in a data extraction form in Microsoft Word (Table 1), which was predesigned and piloted. Two reviewers (DM and GA) extracted data from each report independently. The information on author, year, setting, design, details of the intervention and control, length of follow-up, outcome measures studied and data on effect measures (such as mean and SD in each group postintervention), including statistical significance of change in outcome measure, were extracted. Only those findings relevant to adolescents regarding changes in their SRH outcomes were reported. The accuracy and clarity of the extracted data were checked by two reviewers (TS and PP).

**Table 1.** Data extraction table

Reference Country	Study aim	Study design/study setting	Population	Intervention	Theoryused	Comparison	Follow-up	Outcome explored/tools used	Study findings	
1. Valizadeh et al. (2016)^30^ Iran	Transmitting knowledge to adolescent girls through their mothers about knowledge and practices toward puberty hygiene in Tabriz, Iran	RCT High schools in Tabriz, Iran	364 adolescent girls (aged 11–14 y) and their biological mothers	In addition to the usual care or delivering an educational package to the adolescent girls, their mothers received a 30-min lecture on puberty hygiene in schools, a booklet and two pamphlets (n=120). Following the intervention, students’ knowledge and practices on menstrual health hygiene were examined	Not given	The adolescent girls received the usual care or an educational package (n=124). No intervention was delivered to mothers of the girls	8 wk	Adolescent girls’ knowledge of menstrual hygiene: a validated 15-item questionnaire. Adolescent girls’ practices on menstrual hygiene: a 32-item questionnaire	Mean (SD)	**Baseline** Inter=7.9 (2.0) Contr=8.2 (2.0) **Follow-up** Inter=9.5 (1.7) Contr=9.8 (1.8) **MD**=-0.1 (-0.8–0.4), p=0.859 No sig. diff. between groups **Baseline** Inter=91.3 (10.2) Contr=91.7 (9.8) MD=-0.4 (-3.5—2.6) **Follow-up** Inter=97.2 (11.3) Cont=91.0 (10.5) **MD**=-1.6 (-5.4–2.1), p=0.640
						No-intervention group (n=120)	8 wk	Students’ knowledge on menstrual hygiene: a validated questionnaire. Girls’ practices on menstrual hygiene : a 32-item questionnaire	Mean difference and 95% CI	**Baseline** Inter=7.9 (2.0) Contr=8.5 (2.5) MD=-0.5 (-1.1–0) **Follow-up** Inter=9.5 (1.7) Contr=9.1 (2.4) MD=0.7 (0.0–1.3) **p=0.026** **Baseline** Inter=91.3 (10.2) Cont=93.7 (9.8) **Follow-up** Inter=97.2 (11.3) Cont=97.8 (10.5) -0.6 (-3.0–4.3) p=0.966
2. Florence and Juliana (2020)^51^ Nigeria	Effect of peer- and parent-led educational intervention on HIV knowledge and attitudes of in-school adolescents in selected secondary schools in Ogun State, Nigeria	Quasi-experimental design Selected schools in Ogun state	220 adolescent participants aged 10–19 y and their parents	The adolescent participants were educated on HIV through parents. Parents were trained for 1 wk on HIV prevention by a parent-led group (n=55)	Not given	No-intervention group (n=55) Peer-led group (students were trained by their trained peers on HIV prevention) (n=55)	8 wk	Adolescent participants’ awareness of HIV/AIDS A questionnaire was developed and validated to assess adolescent participants’ knowledge and attitudes on HIV	Mean and SD Mean difference	**Baseline** Parent-led=12.11±2.529 Peer-led=13.11±2.386 Control=10.42±3.430 **Follow-up** Parent-led=20.05±1.533 Peer-led=18.78±1.883 Control=9.71±3.270 **Mean diff**. Parent-led=7.945 (p<0.01) Peer-led=5.673 (p<0.01) Contr=-0.709 (p<0.01)
								Adolescent participants’ attitudes of HIV/AIDS		**Baseline** Parent-led=14.64±3.262 Peer-led=16.11±4.246 Contr=17.27±5.020 **Follow-up** Parent-led=24.84±3.5 Peer-led=21.94±3.233 Contr=14.11±3.971 **Mean diff**. Parent-led=10.2 (p<0.01) Peer-led=5.800 (p<0.01) Contr=-3.164
3. Orak and Okanli (2020)^40^ Turkey	The effect of preventive psychosocial interventions directed towards mothers and children on children's knowledge about protection from sexual abuse	Quasi-experimental study Primary schools in Turkey	80 students aged 9–10 y and their mothers	Children participated in a good touch, bad touch curriculum test (pretest) Two intervention groups: 1. Mother psychoeducation group (Group A) Psychoeducation intervention was offered only to the mothers (n=20 mothers, 20 children) 2. Mother-child psychoeducation group (Group B) Psychoeducation was offered to both mothers and children (23 mothers, 23 children) 3. Only the children received psychoeducation (Group C) (n=23) Mothers were educated about mothers’ role in SA prevention. Children were educated on identifying SA, what to do, etc.	-	No-intervention group included 28 mothers and 29 children (Group D)	1 mo	Knowledge of sexual abuse among children tool: ‘Good Touch Bad Touch Curriculum Test’	Mean and SD	**Baseline** Mother-psychoedu=6.55±1.317 Mother-child psychoedu=7.35±1.424 Children psychoedu=7.48±1.410 Control=7.31±1.312 **Follow-up** Mother-psychoedu=8.15±1.182 Mother-child psychoedu=9.75±0.444 Children psychoedu=9.78±0.518 Control=8.24±0.872 p<0.000 (B>A,D), (C>B,D)
4. Bell et al. (2008)^39^ South Africa	Building protective factors to offset sexually risky behaviors	RCT (cluster randomized trial) Schools in KwaDedangendlale, South Africa	9–13 y-old children (n=557) and their parents	Adaptation of CHAMP-US to South Africa. 10–90 min sessions delivered by trained community caregivers to the families over 10 weekends. The sessions were designed to increase HIV knowledge and decrease the stigma surrounding HIV infections. To increase parenting practices and communication skills of the parents.	TTI	The existing school-based HIV prevention curriculum served as the control condition (n=233)	12 wk	HIV transmission knowledge/ AIDS transmission knowledge scale	Mean (SD)	**Follow-up** Intervention group=0.88 Control group=0.12 Pooled SD=1.54 p<0.0074; effect size=0.5
								Stigma toward HIV/ stigma scale	Mean (SD)	**Follow-up** Intervention group=3.96 Control group=-0.25 p<0.0045; pooled SD=6.03 effect size=0.7
								Frequency of discussing sex with caregivers/ Hard-to-Talk-About scales	Mean (SD)	**Follow-up** Intervention group=2.24 Control group=0.93, pooled SD=5.42 p=0.018; effect size=0.24
								Comfort of discussing sex with caregivers. Hard-to-Talk-About scales	Mean (SD)	**Follow-up** Intervention group=2.08 Control group=1.58 p=0.0651; pooled SD= 6.48; effect size=0.08
5. Shato et al. (2021)^31^ Uganda	Family EE, family social support and sexual risk-taking behaviors among adolescents living with HIV in Uganda: the Suubi+Adherence study	Cluster randomized clinical trial Health centres or clinics in South Uganda	702 adolescents (306 boys, 396 girls) living with HIV (on ART) aged 10–16 y and their caregivers	Standard of care + family EE intervention delivered to 19 clinics (344 participants). Family EE consisted of a child development account, four 1-h group sessions providing financial literacy and small business development training, and mentorship (financial planning, business development and avoiding risk-taking behaviors)	Asset theory Social support theory	Received standard of care (n=20 clinics, 358) medical support and psychosocial support, adherence counseling and monitoring, and family support, delivered by community health work	24 mo	Sexual risk-taking attitude	Mean and SD	Baseline: Inter:1.86 (1.09) Contr: 1.95 (1.13) p=0.29 −0.42 (−0.56, −0.29) (p<0.001)
6. Bogart et al. (2013)^38^ South Africa	Let's Talk!, a South African worksite-based HIV prevention parenting program	RCT worksites in the Western Cape Province	66 parents of adolescents aged 11–15 y and their children	The intervention consisted of five weekly 2-h group sessions for parents (n=34)	Social learning theory, theory of reasoned action, health-belief model	Wait-list control group (n=32)	2 mo	Comfort talking to parents about HIV and sex 16-item scale	Mean and SD	**Baseline** Interv:4.1 (1.9) Control:3.7 (1.9) **Follow-up** Inter:4.6 (1.8) Control:4.2 (1.8) p=0.6 Effect size=0.1
								Number of sex and HIV topics discussed		**Baseline** Interv:7.2 (4.7) Control:9.4 (4.0) **Follow-up** Inter:9.2 (4.8) Control:9.2 (5.1) p=0.24 Effect size=0.3
								Number of new sex and HIV topics discussed		**Follow-up** Inter:3.5 (3.3 Control:2.6 (2.7) p=0.66 Effect size=0.1
7. Gesualdo et al. (2023)^50^ Haiti	Inclusion of parents in a sports-based HIV prevention program for Dominican youth	Quasi-experimental study In schools	Youth participants aged 13–17 y and their parents (n=42)	A single-session workshop for parents on STI prevention of youth (n=17)	Social cognitive theory	No-parents’ session (n=25)	1 mo	Self-efficacy to prevent STI STIs and HIV prevention scale	Mean and SD	**Baseline** Inter:36.71 (±4.61) Control:35.72 (±6.01) **Follow-up** Inter:44.18 (±4.79) Control: 38.84 (±5.51), p=0.05
								Safe sexual behaviors (safe sex behavior questionnaire)		**Baseline** Inter:73.00 (±8.72) Control:69.25 (±4.99) **Follow-up** Inter:84.33 (±2.52) Control:79.50 (±4.80), p=0.18
								Parent-child communication about sex The Parent–Teen Sexual Risk Communication Scale (PTSRC-III)		**Baseline** Inter:18.53 (±7.00) Control: 22.72 (±5.98) **Follow-up** Inter:27.47 (±5.58) Control:21.08 (±5.31)
8. Ismayilova et al. (2011)^36^ Uganda	Family support as a mediator of change in sexual risk-taking attitudes among orphaned adolescents in rural Uganda	Cluster randomized design in primary schools	Suubi included 283 AIDS-orphaned youth aged 11–17 y and their primary caregivers	Adolescents and their primary caregivers received the Subbi intervention (economic empowerment intervention), which included a matched savings account for children, 12 1-h workshops on financial education, monthly peer mentorship sessions for youth (n=137)	Resilience theoretical framework	Control condition: received the *usual care* for orphaned children (n=146)	20 mo	Sexual risk-taking attitudes	Mean and 95%CI Point estimate (beta) and SE	**Follow-up (24 mo)** Reduction in sexual risk-taking attitudes among adolescents Beta=-0.47 (0.13), p<0.05
9. Armistead et al. (2014)^37^ Cape Town, South Africa	Preliminary results from a family-based HIV prevention intervention for South African youth	RCT	Parent-adolescent dyads; female parent and their 10–14-y-old child (n=90)	Six-session intervention for parents to empower them to communicate about sex with children (n=58)	Social learning theory	Wait-list control (n=38)	6 mo	No. of sex topics discussed 8-item scale	Mean (SD)	**Baseline** Inter=4.44 (2.48) Contr=4.79 (2.47) **Follow-up** Inter=5.37 (2.65) Contr**=**5.28 (2.95) Effect size=0.03
								Youth perceived parent responsiveness to sex communication 22-item scale	Mean (SD)	**Baseline** Inter=1.09 (0.34) Contr=1.12 (0.4) **Follow-up** Inter=1.24 (0.4) Contr**=**1.08 (0.32) Effect size=0.44

ART: antiretroviral therapy; EE: economic empowerment; psychoedu: psychoeducation; RCT: randomized controlled trial; SA: sexual abuse; STI: sexually transmitted infection; TTI: theory of triadic influence.

### Study outcomes

The outcomes of interest for this systematic review were adolescents’ knowledge regarding SRH, adolescents’ attitudes toward SRH, SRH practices/behaviors, SRH communication and self-efficacy to prevent sexual risks.

### Risk of bias assessment

The study quality of randomized trials was assessed using the revised Cochrane Risk of Bias tool (ROB-2),^44^ while the ROBINS-I tool^45^ was employed to assess the quality of non-randomized controlled trials and quasi-experimental studies. The ROB-2 tool consisted of five domains, which assessed the risk of bias arising from the randomization process, assignment to intervention, missing outcome data, measurement of outcome and selection of the reported results. The ROBINS-I tool consisted of seven domains that assessed the risk of bias arising from confounding, intervention classification, selection of participants into the study, deviation from intended interventions, missing data, outcome measurement and selection of the reported results.

Study quality was assessed by two independent reviewers (DM and TS). If there was disagreement between reviewers' judgments, third-party intervention (PP) was sought to reach a consensus.

### Data synthesis

Study interventions were broadly categorized into knowledge, attitudes and practices regarding SRH, SRH communication and self-efficacy to prevent sexual risks. The results have been primarily reported using a narrative synthesis of data that reported how the data were presented, a summary of findings and limitations of the synthesis. However, where possible, we conducted a meta-analysis when two or more estimates were available for a given outcome, as mentioned above. The quantitative results were combined using a random-effects model as there was potential methodological heterogeneity between studies (such as different study designs, diverse populations, intervention settings and follow-up). Heterogeneity was summarized using the estimate of Cochran's Q test, and the proportion of variability in effect estimates due to between-study heterogeneity, was summarized using the I^2^ statistic. Individual estimates were pooled using an inverse variance approach and are reported as standardized mean differences (SMDs) with respective 95% CIs using the DerSimonian and Laird methods.^46^ A 95% prediction interval for the random-effects model was calculated if at least three studies were available in a meta-analysis.^47^ SMDs were directly estimated from the postintervention mean and SD for a given study. For cluster trials, the sample size in each group was adjusted when included in the meta-analysis to handle the unit of analysis issue, as suggested in the *Cochrane Handbook*.^33^ We did not assess publication bias for a specific outcome as the number of included studies in any meta-analysis undertaken was <10.^48^

We initially planned to report outcomes by study design (randomized controlled trials [RCTs] and quasi-experimental studies), population type (e.g. female and male adolescents) and age groups in subgroup analyses; however, due to the limited data, we were unable to conduct any further analysis.

## Results

### Summary of studies identified

**Figure 1.**
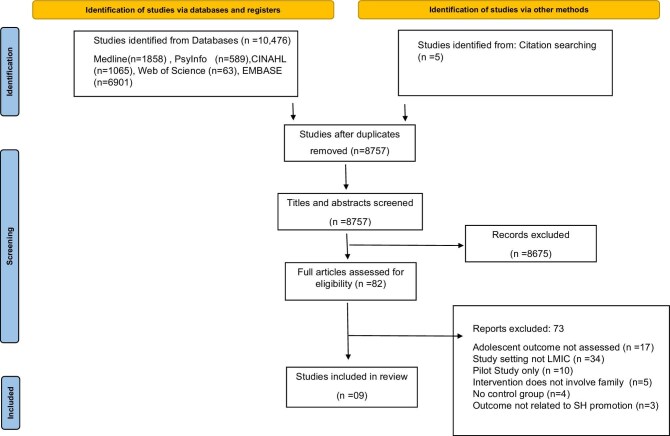
PRISMA flow diagram of the study selection (source^49^). LMIC: low- and middle-income country; PRISMA: Preferred Reporting Items for Systematic Reviews and Meta-Analyses; SH: sexual health.

A total of 8752 unique papers were identified from database searches, of which nine studies were included in the review. The PRISMA flow diagram^49^ indicates the study identification and selection to include in the final review (Figure 1). The inter-rater agreement between two reviewers (DM and GA) for abstract screening was moderate with two reviewers agreeing upon 8705 articles (kappa=0.65).

### Study characteristics

Study characteristics and findings for the nine included studies are summarized in Table 1. Three out of nine included studies were conducted in South Africa,^37–39^ two in Uganda,^31,36^ and one each in Haiti,^50^ Iran,^30^ Turkey^40^ and Nigeria.^51^ Six studies employed RCTs, with two using individual randomization^37,38^ and four using cluster randomization.^30,31,36,39^ Three studies used a quasi-experimental design.^40,50,51^

In this review, 2246 adolescent participants and their families were involved in the analysis of studies included. Five studies focused on early adolescents (aged 10–14 y),^30,37–40^ while four studies involved both early and late adolescents (aged 15–19 y).^31,36,50^

### Interventions

All nine interventions specifically targeted the primary caregivers of adolescents, including biological parents, adopted/foster parents, grandparents, other male or female relatives or an older sibling. Three of the interventions involved only the female caregiver of adolescents,^30,37,40^ while six recruited caregivers irrespective of gender.^31,36,38,39,50,51^ The included interventions incorporated a blend of educational and skill-building activities and varied from single to multiple group sessions.

Six studies employed multiple group sessions,^31,36–39,51^ while three interventions were delivered as a single session. These sessions were either sessions for parents only,^30,38,51^ separate sessions for parents and youth,^30,50^ combined sessions for parents and youth,^31^ or a combination.^37,40^ Program setting varied in interventions. Most of the interventions (n=6) were conducted in the school setting,^30,36,39,40,50,51^ while the others were community-, clinic- and worksite-based sessions.^38^

Seven studies mainly focused on caregiver awareness and skill building to deal with adolescent sexual health concerns.^30,37–40,50^ Two of the studies employed a family economic empowerment intervention to improve sexual health outcomes among adolescents.^31,36^ All interventions employed a face-to-face delivery mode. The trials applied various methods to meet the objectives, such as lecturing and presentation, role-playing, homework assignments, small group discussions, modeling, experiential exercising, skill practice, interactive activities and distribution of booklets.

All trials included a control group that received either the standard of care,^31,36,39^ a different intervention^30^ or no intervention.^30,38–40,50^

### Theoretical frameworks used to develop family interventions

Theory of triadic influence (TTI),^39^ social cognitive theory,^50^ social learning theory,^37,38^ theory of reasoned action^38^ and the health belief model^38^ were the theories adopted by the interventions to increase caregiver awareness and skills to protect their children from sexual risks.

Bell et al. used TTI to guide the CHAMPSA program.^39^ The seven community field principles in CHAMPSA were derived from TTI. During the development of the “Let's Talk!” program,^38^ parent's perception of their child's risks and their confidence in talking to their children about the risks (health belief model), and how children observe, imitate and model their parents (social learning theory), were taken into consideration.

Two family economic empowerment interventions employed social support theory, asset theory and a resilience theoretical framework when developing the interventions.^31,36^ Resilience theory suggests that resources can balance the effects of risks in the environment. Suubi intervention was designed within assets theory, explaining that possession of assets increases adolescents’ hopes and beliefs about the future, reducing their tendency to engage in risky behaviors.^31^ Also, the positive association between adolescents and their caregivers acted as a protective factor, one which would reduce sexual risks among adolescents.

Social cognitive theory and social learning theory were among the most used theories used when designing family-based sexual health interventions. Some trials used constructs from multiple theories.^38^

### Study outcomes

Four studies assessed the effectiveness of family-based interventions in improving adolescents’ sexual health knowledge,^30,39,40,51^ while four assessed their effectiveness in improving adolescents’ attitudes.^31,36,39,51^ Two studies examined the impact of the intervention in improving sexual risk behaviors among youth.^30,50^ Four studies explored the intervention's effect on parent–child sexual communication.^37–39,50^ One study^31^ explored adolescents’ self-efficacy to prevent sexual risk.

### Quality of studies

Apart from two studies^30,39^ that had some concerns over Risk of Bias assessment, all the other studies included in the review had an overall low risk of bias according to the Cochrane ROB tool.

**Figure 2.**
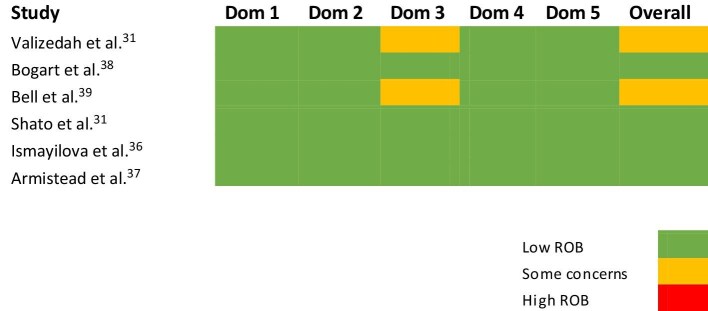
Risk of bias analysis of randomized controlled trials using the revised Cochrane ROB tool for randomized trials. Dom: Domain; ROB: risk of bias.

All six randomized controlled studies included in the review had a low risk of bias for Domain 1: Randomization process. All studies had a low risk of bias for Domain 2: Effect of assignment to intervention. Two studies^26,32^ had some concerns for Domain 3: Risk of bias due to missing outcome data. Although two of the studies had some concerns regarding Domain 4: Risk of bias in measurement of outcome, all studies had a low risk of bias for Domain 5: Risk of bias in selection of the reported results (Figure 2).

**Figure 3.**
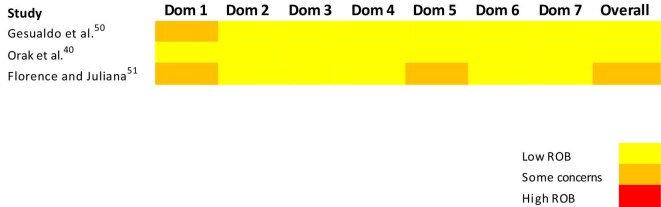
Risk of bias analysis of non-randomized studies using the ROBINS-I tool. Dom: Domain; ROB: risk of bias.

Out of three quasi-experimental studies, two^40,50^ had a low risk of bias, while one^51^ had some concerns over risk of bias assessed using the ROBINS-I tool. All three studies had a low risk of bias for Domain 2: The risk of bias arising from the measurement of the exposure, Domain 3: The risk of bias in the selection of participants into the study, Domain 4: The risk of bias due to postexposure interventions and Domain 7: The risk of bias in the selection of the reported result. Two of the three quasi-experimental studies^50,51^ had some concerns regarding Domain 1: Risk of bias due to confounding, while one study^51^ had some concerns over Domain 6: Risk of bias arising from outcome measurement (Figure 3).

### Effectiveness of family-based interventions on improving adolescent sexual health outcomes

**Figure 4.**
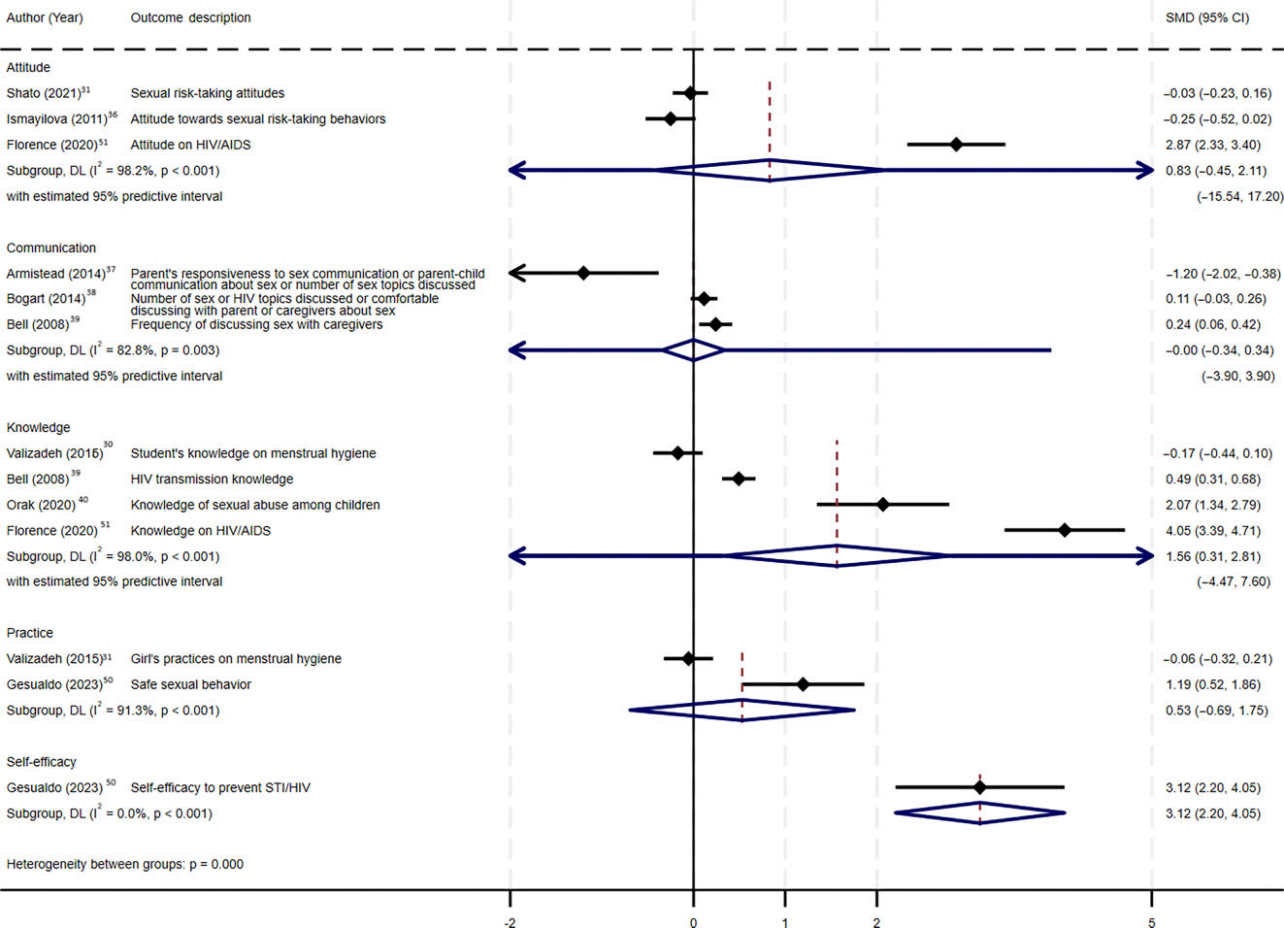
Forest plot showing the effectiveness of each outcome in a meta-analysis. DL: DerSimonian and Laird; STI: sexually transmitted infection.

All nine studies were included in the meta-analysis. The forest plot of the analysis is shown in Figure 4.

The forest plot (Figure 4) presents the results of the meta-analysis on five different outcomes: knowledge, attitudes, communication, practices and self-efficacy. Four studies, which included 1271 participants, were analyzed to determine the effectiveness of family intervention on improving sexual health knowledge among adolescents,^30,39,40,51^ and indicated it had a significant impact on improving adolescents' knowledge in SRH (SMD 1.56, 95% CI 0.31 to 2.81, p=0.014). Three of the above studies^30,39,51^ were categorized as having some risk of bias as per the Cochrane ROB-1 and ROBINS-I tools. Hence, the results should be interpreted with caution.

Analysis of three studies^31,36,51^ that included 1255 participants indicated that family interventions were not significantly effective at changing adolescents’ SRH attitudes (SMD 0.83, 95% CI -0.445 to 2.105, p=0.202).

The pooled effect of the two studies^30,50^ that examined adolescents’ SRH practices indicated that the effectiveness of family-based interventions in changing adolescents' SRH practices was not significant (SMD 0.53, 95% CI -0.69 to 1.75, p=0.395).

Three studies^37–39^ (n=983) assessed the effectiveness of a family-based sexual health intervention to improve adolescents' communication with their families about sex, suggesting they might have different measures. The results indicated that family interventions had no significant impact on improving the SRH communication of adolescents with their families (SMD 0.001, 95% CI -0.34 to 0.34, p = 0.997).

Further, only one study^50^ compared the self-efficacy to prevent STIs/HIV, showing high intervention effectiveness (SMD 3.13, 95% CI 2.20 to 4.05, p<0.001).

All meta-analysis results are subject to high between-study heterogeneity as I^2^ statistics ranged from 82.8 to 98.2%, and prediction intervals often cross the line of no effect. Therefore, the results should be interpreted with caution. However, this heterogeneity is mainly caused by the variations in the measurement/tool used across the studies.

### Impact of engaging parents in delivering sexual health interventions to adolescents

Valizadeh et al.^30^ showed that educating mothers and transferring knowledge to daughters, and directly educating daughters effectively, improved adolescent girls’ knowledge about puberty hygiene compared with the no-intervention group. Nevertheless, the findings indicate that neither directly educating adolescents, nor educating them through mothers, would significantly improve the adolescents’ sexual health practices. The study could not elicit a significant difference in the improvement in girls’ knowledge or practices regarding puberty hygiene between mothers’ vs girls’ education. However, the validity of the intervention carried out in the study is questionable because it was limited to a 30 min and few information, education, and communication (IEC) materials. In addition, the follow-up time (8 wk) was not adequate to expect an attitudinal or behavioral change in adolescents.

Florence and Juliana^51^ showed that parent-led interventions have a greater effect on increasing adolescents' knowledge about HIV prevention compared with peer-led interventions. The same study also indicated that the parent-led intervention group had a greater impact on the adolescents’ attitudinal disposition. The results implied that adolescents were more receptive to sexual health information when delivered by a parent figure.^51^ In this study, parents underwent a comprehensive 1-wk training session on HIV prevention, which provided them with sufficient knowledge and skills to effectively pass the message on to their offspring.

According to the results of Orak and Okanli,^40^ the adolescents in both the mother–child psychoeducation group and the child psychoeducation group had significantly higher knowledge about sexual abuse prevention at the follow-up compared with the control group and the mother psychoeducation group. The study shows that interventions targeted at parents alone are ineffective in improving adolescent outcomes while stressing the importance of conducting combined interventions targeting parents and children. The latter will facilitate the transfer of sexual health messages from parents to children by reducing the fear and embarrassment of discussing the topic. Similarly, a South African worksite-based parenting program^38^ demonstrated that parent-targeted intervention was not significantly effective in improving adolescents’ communication with their parents on sex concerning comfort, the number of sex topics discussed and the number of new topics discussed. However, as per parent reports, a significant improvement was seen in the comfort of talking to the child about sex and the number of sex topics discussed. The findings demonstrate that although parenting intervention may have a significant impact on parent-perceived skills and awareness about discussing sexual health matters with children, the transmission of knowledge to the children may not be as effective as expected. The finding further highlights the importance of conducting combined interventions targeting both parties, adolescents and their parents, to elicit better results. The study by Gesualdo et al.^50^ found that parental involvement was beneficial in reinforcing family dialogues and, thereby, increasing parent–child communication of SRH, adolescents’ self-efficacy to prevent STIs and safe sexual behaviors. Similarly, Bell et al.^39^ demonstrated that strengthening family dynamics can effectively enhance individual and community protective factors for sexual health. The findings of Armistead et al.^37^ suggested that engaging families in HIV prevention efforts for young people was effective in South Africa. The program was effective in improving family communication, increasing HIV knowledge and encouraging safe sexual practices among youth. The results imply that involving families can be a promising strategy for HIV transmission risk in the South Asian context.

The findings of the ‘Let's Talk!’ intervention targeted at parents alone^38^ indicated that the intervention was not effective in improving child outcomes as perceived by the child, such as comfort in talking to parents about sex and the number of sexual topics discussed. However, parents’ reports suggested that the intervention improved their communication skills and comfort level, even although these changes did not translate into measurable benefits to children.

The Suubi+Adherence study^31^ examined the impact of family economic empowerment intervention and social support in improving sexual risk-taking behaviors among youth living in Uganda. The study suggested that saving programs and financial literacy training, along with increased family support, can reduce engagement in risky sexual behaviors among youth. Similarly, Ismayilova et al.^36^ highlighted that family support plays a crucial role in shaping adolescents’ attitudes toward sexual health, particularly by reducing sexual risk-taking behaviors. The study showed that adolescents who received a higher level of family support had a more positive attitude towards safe sex behaviors, suggesting interventions that strengthen family ties are effective for sexual risk reduction in the adolescent population.

## Discussion

The findings of this systematic review and meta-analysis provide valuable insights into the effectiveness of family-based sexual health interventions for adolescent sexual health promotion in LMIC settings. The findings reveal a mixed picture of the impact of family-based interventions, with significant improvement seen in the sexual health knowledge of adolescents. By contrast, the improvement in sexual health attitudes, sexual communication or sexual behaviors and practices was limited or insignificant (Figure 4). Findings suggest that changing long-standing beliefs, norms and practices may need a multifaceted approach beyond family efforts. However, because most of the interventions in the review had short follow-up periods, long-term interventions may be necessary to change parents’ beliefs. This change could then be transferred to their offspring, potentially leading to improved attitudes and behaviors in future generations. In contrast to the above findings, a review that was carried out to explore parent- and family-based intervention effectiveness on sexual health outcomes in young people in the UK and the USA reported that parent-based interventions were effective at reducing adolescents’ sexual risk-taking behaviors.^24^ Adding to this, a review by Widman et al.^26^ reported that parent-based interventions significantly increase condom use among adolescents and promote parent–adolescent sexual communication. However, the same review also reported that parent-based interventions have no significant impact on delaying sexual activity among adolescents.

Furthermore, the findings of the present review indicated that multisession interventions had a high level of attention and retention among participants and might be more effective than single-session interventions when improving family sexual communication.^37^ However, this finding is in contrast to a previous review^52^ that showed parent-based programs were effective in improving adolescent sexual health outcomes irrespective of the intervention dose. In addition, several studies in the review suggested that the intervention effect tends to diminish over extended follow-up periods. This highlights the importance of incorporating booster sessions following the initial program to sustain the intervention effect over time.^37^ For instance, a study by Pedlow and Carey^53^ found that interventions supplemented with booster sessions can significantly reduce sexual risk behaviors in adolescents.

Interventions that included adolescents and their family members^36,40^ showed improved sexual health outcomes compared with those that targeted parents or adolescents alone.^30,38,50^ It was observed that including both caregivers and adolescents in the same program facilitated open communication and a better understanding of sexual health topics.

Four out of nine studies included in the review focused exclusively on female caregivers. This may reflect the predominant role that female guardians play as primary caregivers in many cultures across LMICs. Supporting this finding, the research found that many adolescents prefer to share sexual health matters with their mothers rather than their fathers.^54,55^

Besides, some of the studies in the present review directly adopted an intervention previously shown to be effective in US- or UK-based samples. For example, the CHAMP family program, originally developed in the USA, was adapted for use in South Africa under the CHAMPSA initiative.^39^ Similarly, the ‘Let's Talk!’ intervention was adapted from ‘Talking Parents - Healthy Teens’, a successful US-based program that improved family sex communication. The adaptation retained the core components of the original program, which may not be appropriate for a different cultural context. This may have reduced the expected effectiveness of the program. The available literature demonstrates the importance of tailoring interventions to increase cultural appropriateness, relevance and societal acceptability to bring effectiveness.^56,57^

It was noted that most of these studies were conducted in the African region, while some were from the Middle East and Latin American context. It was noteworthy that no such interventions to assess the impact of family-based interventions on adolescent sexual outcomes were conducted in Asian settings, particularly from the eastern and southern parts of the continent. This may be attributed to the taboos and stigma surrounding the implementation of sexual health programs in this part of the world.^58^ With the increasing rates of sexual activity, sexual violence and STIs in Southeast Asia, the review identified a clear gap in exploring new strategies to promote the sexual health of young people in the region.

Although this review explored interventions targeted at the family, including parents, siblings or any other family members, we only encountered studies that assessed interventions targeted at the primary caregiver or the parent of the adolescent. None of the interventions that focused on promoting adolescent sexual health outcomes involved siblings. This may be attributed to the differential influence and relationships between siblings. Evidence suggests that the nature and impact of sibling relationships can vary depending on the birth order, age spacing, gender or the quality of interaction.^59^ The challenge of controlling the effect of heterogeneous relationships may have paused the designing of sibling-targeted interventions. Adding to the above, previous literature shows that parent-based interventions had a greater impact on parent–child communication than when the intervention was targeted at other family members.^24^

The mixed effectiveness of the interventions included in the review suggests that not all strategies are equally successful in improving adolescent sexual health outcomes across LMICs. Careful selection of intervention approaches to suit the sociocultural and economic context of the populations is important.

### Strengths and weaknesses

A sound methodological approach used to synthesize available literature and use all available interventions, including randomized, non-randomized and quasi-experimental studies in the meta-analysis, was a strength of this study. Besides, having two reviewers to conduct article screening and quality assessment improved the quality of the review. Not having any studies with a high risk of bias as per the Cochrane ROB-1 or ROBINS-I tools improved the confidence of the review findings. Apart from three studies, all other studies included in the review had low risk of bias, which is a strength of this review. The inclusion of a narrative review along with the meta-analysis helped in-depth analysis of the studies. Being the first review to focus on this topic in LMICs is an added strength of this study. Despite these strengths, there were some limitations that need attention when interpreting the findings.

High heterogeneity seen in several subgroups (e.g. I^2^=98.2% for knowledge, I^2^=61.5% for communication) raises concern about the variability of the studies included in the meta-analysis. High heterogeneity was created by the variations in intervention designs, cultural contexts, outcome measures and varying participant groups. High heterogeneity can limit the generalizability of findings. High heterogeneity is greatly impacted by the variations in how the outcome measure was defined across the studies. For example, parent–adolescent sex communication was measured as the frequency of communication in some studies, while others assessed the number of sexual topics discussed, communication comfort or parents’ responsiveness to communication. Similarly, when measuring sexual knowledge, one study assessed adolescents’ knowledge about menstrual hygiene, while another study assessed HIV transmission knowledge, or knowledge about sex abuse. Moreover, the variations in the measurement/tool used across studies could obscure the ability to detect the most precise estimate for intervention efficacy.

Database searches were limited to five reputable scientific research databases. The possibility of missing other relevant literature that may have been published in other databases cannot be ruled out. The review only included articles published in English in peer-reviewed journals and did not include evidence from gray literature.

All interventions included in the review were delivered in person. This restricts the interpretation of the results to interventions that use novel technologies. Recent systematic reviews assessing the effectiveness of e-health or mobile health interventions have shown promising results in improving youth sexual health outcomes.^60^

### Conclusion

The studies included in the review reported mixed results of FBSI in improving adolescent SRH outcomes in LMICs. While the FBSI showed promising results in improving adolescent sexual health knowledge, their influence on changing attitudes, practices and family communication around sexual health was inconsistent. The results of the review suggest the importance of conducting combined interventions with the participation of both parents and adolescents. In addition, the importance of conducting multisession interventions and incorporating booster sessions was highlighted. Gaps in culturally tailored interventions, sibling-targeted interventions and studies from the Asian region were emphasized.

### Recommendations

This systematic review and meta-analysis suggest that future interventions should prioritize multisession programs that directly involve adolescents and their primary caregivers to enhance the effective transfer of SRH knowledge among them. Moreover, future programs must include culturally adopted, contextually relevant material to increase the acceptability and effectiveness in LMIC settings. Family-based interventions should integrate components that aim at improving adolescents' attitudes and practices about SRH. Longer follow-up periods and refresher activities are recommended to achieve sustainable changes in attitudes and sexual practices.

Conducting more research in under-represented areas, particularly Asia, is important to identify the best intervention strategies that suit different cultural contexts. Additionally, future research should focus on training parents and adolescents on effective SRH communication strategies. Exploring the involvement of family members other than the primary caregivers, such as siblings and grandparents, in FBSHI, is important to navigate its effectiveness on knowledge transfer and communication.

### Study implications for public health policies

The role of the family should be taken into consideration when developing public health policies to improve adolescent SRH in LMICs. Implementing culturally sensitive parenting interventions parallel to school- or community-based comprehensive sexuality education programs for adolescents will be an effective way to pass sexual health messages to children. Future policies should target empowering and capacity building of the parents and caregivers to actively participate in adolescent sexual health matters.

## Data Availability

Data underlying this article are available in the article and its online supplementary material.

## Annexure 1 Annexure 1 Search String used to retrieve studies on Family-based Sexual health interventions for Adolescents in LMIC setting

Table A1. Explanation of the symbols used in the search strategy.

**Annexure 1:**

MH	Indicates an index term (MeSH heading) (MEDLINE)
DE	Indicates a search term for subject heading (PsyInfo)
exp	Before an index term indicates that all subheadings were selected (EMBACE)
.ab.	Indicates a search for a term in the abstract (MEDLINE/EMBACE)
.pt.	Indicates a search for term in publication type
.sh.	Indicates a search for a term in subject heading
.ti.	Indicates a search for a term in the title (MEDLINE)
.ti,ab,kf	Indicates a search for a term in the title/abstract/word(s) in keyword (MEDLINE/EMBASE)
TS	Indicates Title Search (Web of Science)
.tw	Indicates a search for a term in textwords, i.e. title/abstract (EMBACE/Ovid)
*	At the end of a term indicates that this term has been truncated
#	Mandated wild card character stands for one character within, or at the end of, a word. (EMBACE)
?	Optional wild card character stands for zero or one character within, or at the end of, a word (EMBACE/Ovid)
adj	Search for two terms where they appear adjacent to each other in the same order (EMBACE/Ovid)
adj*n*	Search for two terms where they appear within *n* words of each other (EMBACE/Ovid)
n	Search for two terms where they appear within *n* words of each other (MEDLINE)
W	Search for two terms where they appear within W number of words of each other (Web of Science)

**Annexure 1:** MEDLINE (EBSCO) Searchk

	**Query**	**Results**
S1	(MH "Adolescent") OR (MH "Child")	3 158 155
S2	TI Adolescen* OR AB Adolescen*	351 077
S3	TI youth* OR AB youth*	98 056
S4	TI teen* OR AB teen*	34 466
S5	TI juvenile* OR AB juvenile*	94 722
S6	TI emerg* N2 adult* OR AB emerg* N2 adult*	10 897
S7	TI early N2 adult* OR AB early N2 adult*	15 436
S8	TI ( young N2 (adult* or person or people or woman or women or man or men) ) OR AB ( young N2 (adult* or person or people or woman or women or man or men) )	235 646
S9	TI ( Child* OR boy* OR girl* ) OR AB ( Child* OR boy* OR girl* )	1 789 520
S10	S1 OR S2 OR S3 OR S4 OR S5 OR S6 OR S7 OR S8 OR S9	4 078 944
S11	(MH "Developing Countries")	81 209
S12	TI ( "middle income*" W0 (countr* or nation or nations or econom*) ) OR AB ( "middle income*" W0 (countr* or nation or nations or econom*) )	34 421
S13	TI ( "Low and middle income*" W0 (countr* or nation or nations or econom*) ) OR AB ( "Low and middle income*" W0 (countr* or nation or nations or econom*) )	24 464
S14	TI ( developing W0 (countr* or nation or nations or econom*) ) OR AB ( developing W0 (countr* or nation or nations or econom*) )	73 655
S15	TI ( ("under developed" or underdeveloped) W0 (countr* or nation or nations or econom*) ) OR AB ( ("under developed" or underdeveloped) W0 (countr* or nation or nations or econom*) )	1508
S16	TI ( "3rd world" W0 (countr* or nation or nations or econom*) ) OR AB ( "3rd world" W0 (countr* or nation or nations or econom*) )	10
S17	TI ( "third world" W0 (countr* or nation or nations or econom*) ) OR AB ( "third world" W0 (countr* or nation or nations or econom*) )	1101
S18	TI ( LAMI W0 (countr* or nation or nations or econom*) ) OR AB ( LAMI W0 (countr* or nation or nations or econom*) )	51
S19	TI ( LMIC W0 (countr* or nation or nations or econom*) ) OR AB ( LMIC W0 (countr* or nation or nations or econom*) )	136
S20	TI ( "poor income" W0 (countr* or nation or nations or econom*) ) OR AB ( "poor income" W0 (countr* or nation or nations or econom*) )	5
S21	TI ( "low* income" W0 (countr* or nation or nations or econom*) ) OR AB ( "low* income" W0 (countr* or nation or nations or econom*) )	10 343
S22	TI ( "less* developed" W0 (countr* or nation or nations or econom*) ) OR AB ( "less* developed" W0 (countr* or nation or nations or econom*) )	1506
S23	TI ( "least developed" W0 (countr* or nation or nations or econom*) ) OR AB ( "least developed" W0 (countr* or nation or nations or econom*) )	313
S24	(MH "Asia") OR (MH "Asia, Southern") OR (MH "Asia, Southeastern") OR (MH "Asia, Northern") OR (MH "Asia, Eastern") OR (MH "Asia, Western") OR (MH "Asia, Central")	42 042
S25	(MH "Africa")	33 261
S26	(MH "Caribbean Region") OR (MH "West Indies")	9143
S27	(MH "Central America") OR (MH "South America") OR (MH "Latin America")	24 339
S28	(MH "Europe, Eastern")	4554
S29	TI ( (South or Southern) W0 Asia* ) OR AB ( (South or Southern) W0 Asia* )	13 340
S30	TI ( ("South east*" or Southeast*) W0 Asia* ) OR AB ( ("South east*" or Southeast*) W0 Asia* )	22 964
S31	TI West* W0 Asia* OR AB West* W0 Asia*	1214
S32	TI ( (Middle or Far) W0 East* ) OR AB ( (Middle or Far) W0 East* )	23 175
S33	TI Africa* OR AB Africa*	284 832
S34	TI ( Latin or Central or South) W0 America* ) OR AB ( Latin or Central or South) W0 America* )	56 601
S35	TI ( Caribbean* or "West Indies" ) OR AB ( Caribbean* or "West Indies" )	19 661
S36	TI East* W0 Europe* OR AB East* W0 Europe*	9497
S37	Afghan* or Angola* or Armenia*	21 475
S38	Azerbaijan or Afghanistan or Algeria or Angola	27 235
S39	Bangladesh* or Benin* or Bhutan*	54 803
S40	Bolivia* or "Burkina Faso*" or "Upper Volta*"	14 648
S41	Burundi* or Brazil or Belarus or Bulgaria or Bhutan or Benin or Bosnia	563,704
S42	Botswana or Brazil OR Burkina	523,064
S43	"Cabo Verd*" or "Cape Verd*"	995
S44	Cambodia* or Cameroon* or "Central African Republic*”	24 485
S45	Chad* or Comoros or Comorian or Congo*	49 728
S46	"Cote d'Ivoire" or Colombia or China	2 881 832
S47	"Ivory Coast" or Ivorian*	2405
S48	Djibouti* or “Democratic Republic of the Congo” or Dominica OR “Dominican Republic”	11 972
S49	Ecuador	15 325
S50	Egypt* or "El Salvador*"	146 253
S51	Eritrea* or Ethiopia*	42 926
S52	Gambia* or Georgia* or Ghana* or Guatemala*	34 942
S53	Guinea* or Guyan* or Gabon or Guinea-Bissau	177 568
S54	Haiti* or Hondura*	8790
S55	India* or Indonesia* or Iran or Iraq or Jordan*	1 467 293
S56	Kenya* or Kiribati* or Korea* or Kosov* or Kyrgyz*	45 168
S57	Lesotho or Mosotho or Basotho	1240
S58	Liberia* or Lao*	26 705
S59	Madagasca* or Malagasy	8074
S60	Malawi* or Mali or Mali's or Malian* or Mauritania* or Maldives	22 994
S61	Micronesia* or Moldov* or Mongolia*	31 858
S62	Morocc* or Mozambi* or Myanmar* or Burma* or Burmese*	45 637
S63	Nepal* or Nicaragua* or Niger* or Nigeria or “North Marcedonia”	104 220
S64	Pakistan* or PNG* or Paraguay* or Philippines* or Filipino*	149 424
S65	Rwanda* or Samoa* or "Sao Tome*" or Principe*	19 849
S66	Senegal* or "Sierra Leone*" or "Solomon Island*"	18 633
S67	Somalia* or Sudan* or "Sri Lanka*"	38 738
S68	Swaziland* or Syria* or Tajik*	22 731
S69	Tanzania* or Timor* or Togo* or Tonga*	33 106
S70	Tunisia* or Turk*	448 686
S71	Uganda* or Ukrain*	78 503
S72	Uzbek* or Vanuatu* or Vietnam*	47 245
S73	"West Bank*" or Gaza* or Palestin*	11 794
S74	Yemen* or Zambia* or Zimbabwe*	27 871
S75	S11 OR S12 OR S13 OR S14 OR S15 OR S16 OR S17 OR S18 OR S19 OR S20 OR S21 OR S22 OR S23 OR S24 OR S25 OR S26 OR S27 OR S28 OR S29 OR S30 OR S31 OR S32 OR S33 OR S34 OR S35 OR S36 OR S37 OR S38 OR S39 OR S40 OR S41 OR S42 OR S43 OR S44 OR S45 OR S46 OR S47 OR S48 OR S49 OR S50 OR S51 OR S52 OR S53 OR S54 OR S55 OR S56 OR S57 OR S58 OR S59 OR S60 OR S61 OR S62 OR S63 OR S64 OR S65 OR S66 OR S67 OR S68 OR S69 OR S70 OR S71 OR S72 OR S73 OR S74	6 566 244
S76	(MH "Family") OR (MH "Family Characteristics") OR (MH "Parents+") OR (MH "Family Relations")	256 371
S77	TI ( Family OR Families ) OR AB ( Family OR Families ) )	1 131 990
S78	TI ( mother OR mothers ) OR AB ( mother OR mothers )	254 426
S79	TI ( father OR fathers ) OR AB ( father OR fathers )	47 666
S80	TI ( Parent OR parents ) OR AB ( Parent OR parents )	326 723
S81	TI “Primary caregiver” OR AB “Primary caregiver”	1659
S82	TI “Primary care giver” OR AB “Primary care giver”	87
S83	TI "home based" OR AB "home based"	15 023
S84	TI "at home" OR AB "at home"	130 560
S85	TI "in the home" OR AB "in the home"	18 343
S86	S76 OR S77 OR S78 OR S79 OR S80 OR S81 OR S82 OR S83 OR S84 OR S85	1 766 979
S87	(MH "Sexual Health") OR (MH "Reproductive Health") OR (MH "Reproductive Health Services+") OR (MH "Sexuality+") OR (MH "Sexual Trauma+") OR (MH "Sexual Behavior+") OR (MH "Sexual Harassment") OR (MH "Reproductive Rights")	172 159
S88	TI ( condom or “safe sex” or condoms ) OR AB ( condom or “safe sex” or condoms )	21 219
S89	TI ( (OC or Contraceptive) N2 pill OR (oral N2 contraceptive) ) OR AB ( (OC or Contraceptive) N2 pill OR (oral N2 contraceptive) )	24 972
S90	TI ( abstain or abstinen* ) OR AB ( abstain or abstinen* )	31 112
S91	TI sex* N2 intercourse OR AB sex* N2 intercourse	10 899
S92	TI ( pregnan* N2 (adolescen* or teen* or schoolchild*) ) OR AB ( pregnan* N2 (adolescen* or teen* or schoolchild*) )	7596
S93	TI ( pregnan* N3 (prevent* or interrupt* or unplanned or unwanted or mistimed) ) OR AB ( pregnan* N3 (prevent* or interrupt* or unplanned or unwanted or mistimed) )	17 812
S94	TI ( sexual* W0 transmi* or STI or STIs or STD or STDs or venereal ) OR AB ( sexual* W0 transmi* or STI or STIs or STD or STDs or venereal )	57 319
S95	TI ( hiv or hiv‐1* or hiv‐2* or hiv1 or hiv2 or "human immunodeficiency virus" or "human immunedeficiency virus" or "human immuno‐deficiency virus" or "human immune‐deficiency virus" or ((human W0 immun*) AND (“deficiency virus")) or "acquired immunodeficiency syndrome" or "acquired immunedeficiency syndrome" or "acquired immuno‐deficiency syndrome" or "acquired immune‐deficiency syndrome" or ((acquired W0 immun*) AND (“deficiency syndrome" ) OR AB ( hiv or hiv‐1* or hiv‐2* or hiv1 or hiv2 or "human immunodeficiency virus" or "human immunedeficiency virus" or "human immuno‐deficiency virus" or "human immune‐deficiency virus" or ((human W0 immun*) AND (“deficiency virus")) or "acquired immunodeficiency syndrome" or "acquired immunedeficiency syndrome" or "acquired immuno‐deficiency syndrome" or "acquired immune‐deficiency syndrome" or ((acquired W0 immun*) AND (“deficiency syndrome" )	422 473
S96	TI ( ( antiretroviral* or anti‐retroviral* or ARV*) N2 (complian* or adheren*) ) OR AB ( ( antiretroviral* or anti‐retroviral* or ARV*) N2 (complian* or adheren*) )	4109
S97	TI ( (hpv or papilloma virus* or papillomavirus*) N2 (vaccinat* or revaccinat* or immuniz* or immunis* or immunother* or inoculat* or innoculat* or prophyla*) ) OR AB ( (hpv or papilloma virus* or papillomavirus*) N2 (vaccinat* or revaccinat* or immuniz* or immunis* or immunother* or inoculat* or innoculat* or prophyla*) )	9406
S98	TI ( (sexual or spous* or “intimate partner”) N3 (violen* or abus* or rape) ) OR AB ( (sexual or spous* or “intimate partner”) N3 (violen* or abus* or rape) )	34 039
S99	TI ( pubert* or pubescen* ) OR AB ( pubert* or pubescen* )	45 847
S100	TI ( menstruat* or menstrual* ) OR AB ( menstruat* or menstrual* )	53 508
S101	TI ( abort* or miscarr* or (pregnan* N2 terminat*) ) OR AB ( abort* or miscarr* or (pregnan* N2 terminat*) )	107 415
S102	TI ( sex* N2 (protected or unprotected or safe or unsafe or risk* or behavio*) ) OR AB ( sex* N2 (protected or unprotected or safe or unsafe or risk* or behavio*) )	59 600
S103	TI ( reproductive N2 (health or care or service*) ) OR AB ( reproductive N2 (health or care or service*) )	21 315
S104	S87 OR S88 OR S89 OR S90 OR S91 OR S92 OR S93 OR S94 OR S95 OR S96 OR S97 OR S98 OR S99 OR S100 OR S101 OR S102 OR S103	908 346
S105	(MH "Clinical Trials as Topic") OR (MH "Pragmatic Clinical Trials as Topic") OR (MH "Controlled Clinical Trials as Topic") OR (MH "Clinical Studies as Topic") OR (MH "Non-Randomized Controlled Trials as Topic")	209 471
S106	TI natural N2 experiment* OR AB natural N2 experiment*	7589
S107	TI Quasi N2 experiment* OR AB Quasi N2 experiment*	21 519
S108	TI Quasiexperiment* OR AB Quasiexperiment*	880
S109	TI interven* N2 stud* OR AB interven* N2 stud*	80 974
S110	TI interven* N2 effect* OR AB interven* N2 effect*	100 706
S111	TI ( Clinical W0 (trial* OR stud*) ) OR AB ( Clinical W0 (trial* OR stud*) )	639 966
S112	TI Comparative W0 stud* OR AB Comparative W0 stud*	122 267
S113	TI ( (multicenter* OR multicentre*) N2 (stud* OR trial*) ) OR AB ( (multicenter* OR multicentre*) N2 (stud* OR trial*) )	133 116
S114	TI ( Controlled N2 (stud* OR trial*) ) OR AB ( Controlled N2 (stud* OR trial*) )	476 091
S115	TI ( (randomised OR randomized) N2 (stud* OR trial*) ) OR AB ( (randomised OR randomized) N2 (stud* OR trial*) )	589 765
S116	TI ( RCT OR nRCT ) OR AB ( RCT OR nRCT )	74 344
S117	S106 OR S107 OR S108 OR S109 OR S110 OR S111 OR S112 OR S113 OR S114 OR S115 OR S116	1 547 976
S118	S10 AND S75 AND S86 AND S104 AND S117	1858

**Table:** PsycInfo (EBSCO) Search

	**Query**	**Result**
S1	DE "Adolescent Behavior" OR DE "Adolescent Development" OR DE "Adolescent Behavior" OR DE "Adolescent Characteristics" OR DE "Adolescent Health" OR DE "Early Adolescence" OR DE "Late Adolescence" OR DE "Adolescent Fathers" OR DE "Adolescent Health" OR DE "Adolescent Mothers" OR DE "Adolescent Pregnancy"	85 846
S2	DE "Adult Offspring"	4619
S3	TI Adolescen* OR AB Adolescen*	273 107
S4	TI youth* OR AB youth*	120 383
S5	TI teen* OR AB teen*	24 565
S6	TI juvenile* OR AB juvenile*	27 747
S7	TI emerg* N2 adult* OR AB emerg* N2 adult*	8639
S8	TI early N2 adult* OR AB early N2 adult*	9024
S9	TI ( ( young N2 (adult* or person or people or woman or women or man or men) ) ) OR AB ( ( young N2 (adult* or person or people or woman or women or man or men) ) )	120 516
S10	TI ( Child* OR boy* OR girl* ) OR AB ( Child* OR boy* OR girl* )	842 932
S11	S1 OR S2 OR S3 OR S4 OR S5 OR S6 OR S7 OR S8 OR S9 OR S10	1 114 994
S12	DE "Developing Countries"	9744
S13	TI ( "middle income*" W0 (countr* or nation or nations or econom*) ) OR AB ( "middle income*" W0 (countr* or nation or nations or econom*) )	5659
S14	TI ( "Low and middle income*" W0 (countr* or nation or nations or econom*) ) OR AB ( "Low and middle income*" W0 (countr* or nation or nations or econom*) )	4430
S15	TI ( developing W0 (countr* or nation or nations or econom*) ) OR AB ( developing W0 (countr* or nation or nations or econom*) )	11 046
S16	TI ( ( "under developed" or underdeveloped) W0 (countr* or nation or nations or econom*) ) OR AB ( ( "under developed" or underdeveloped) W0 (countr* or nation or nations or econom*) )	226
S17	TI ( "3rd world" W0 (countr* or nation or nations or econom*) ) OR AB ( "3rd world" W0 (countr* or nation or nations or econom*) )	13
S18	TI ( "third world" W0 (countr* or nation or nations or econom*) ) OR AB ( "third world" W0 (countr* or nation or nations or econom*) )	260
S19	TI ( LAMI W0 (countr* or nation or nations or econom*) ) OR AB ( LAMI W0 (countr* or nation or nations or econom*) )	62
S20	TI ( "third world" W0 (countr* or nation or nations or econom*) ) OR AB ( "third world" W0 (countr* or nation or nations or econom*) )	260
S21	TI ( LMIC W0 (countr* or nation or nations or econom*) ) OR AB ( LMIC W0 (countr* or nation or nations or econom*) )	30
S22	TI ( "poor income" W0 (countr* or nation or nations or econom*) ) OR AB ( "poor income" W0 (countr* or nation or nations or econom*) )	1
S23	TI ( low* income" W0 (countr* or nation or nations or econom*) ) OR AB ( low* income" W0 (countr* or nation or nations or econom*) )	6820
S24	TI ( less* developed" W0 (countr* or nation or nations or econom*) ) OR AB ( less* developed" W0 (countr* or nation or nations or econom*) )	488
S25	TI ( "least developed" W0 (countr* or nation or nations or econom*) ) OR AB ( "least developed" W0 (countr* or nation or nations or econom*) )	85
S26	DE "Asians"	12 176
S27	DE "African Cultural Groups" OR DE "Caribbean Cultural Groups"	4466
S28	MA "Central America" or "Latin America" or "South America"	977
S29	TI ( (South or Southern) W0 Asia* ) OR AB ( (South or Southern) W0 Asia* )	3207
S30	TI ( ("South east*" or Southeast*) W0 Asia* ) OR AB ( ("South east*" or Southeast*) W0 Asia* )	2696
S31	TI West* W0 Asia* OR AB West* W0 Asia*	114
S32	TI ( (Middle or Far) W0 East* ) OR AB ( (Middle or Far) W0 East* )	4593
S33	TI Africa* OR AB Africa*	91 116
S34	TI ( (Latin or Central or South) W0 America* ) OR AB ( (Latin or Central or South) W0 America* )	10 369
S35	TI ( Caribbean* or "West Indies" ) OR AB ( Caribbean* or "West Indies" )	4760
S36	TI East* W0 Europe* OR AB East* W0 Europe*	3018
S37	Afghan* or Angola* or Armenia*	5565
S38	Azerbaijan or Afghanistan or Algeria or Angola	5073

**Table:**

	**Query**	**Result**
S39	Bangladesh* or Benin* or Bhutan*	5114
S40	Bolivia* or "Burkina Faso*" or "Upper Volta*"	1378
S41	Burundi* or Brazil or Belarus or Bulgaria or Bhutan or Benin or Bosnia	70 241
S42	Botswana or Brazil OR Burkina	66 444
S43	"Cabo Verd*" or "Cape Verd*"	161
S44	Cambodia* or Cameroon* or "Central African Republic*”	2994
S45	Chad* or Comoros or Comorian or Congo*	7438
S46	"Cote d'Ivoire" or Colombia or China	159 806
S47	"Ivory Coast" or Ivorian*	385
S48	Djibouti* or “Democratic Republic of the Congo” or Dominica OR “Dominican Republic”	1528
S49	Ecuador	1669
S50	Egypt* or "El Salvador*"	6752
S51	Eritrea* or Ethiopia*	3432
S52	Gambia* or Georgia* or Ghana* or Guatemala*	6000
S53	Guinea* or Guyan* or Gabon or Guinea-Bissau	5865
S54	Haiti* or Hondura*	2171
S55	India* or Indonesia* or Iran or Iraq or Jordan*	160 174
S56	Kenya* or Kiribati* or Korea* or Kosov* or Kyrgyz*	6258
S57	Lesotho or Mosotho or Basotho	325
S58	Liberia* or Lao*	2332
S59	Madagasca* or Malagasy	752
S60	Malawi* or Mali or Mali's or Malian* or Mauritania* or Maldives	2224
S61	Micronesia* or Moldov* or Mongolia*	2285
S62	Morocc* or Mozambi* or Myanmar* or Burma* or Burmese*	4638
S63	Nepal* or Nicaragua* or Niger* or Nigeria or “North Marcedonia”	11 988
S64	Pakistan* or PNG* or Paraguay* or Philippines* or Filipino*	14 011
S65	Rwanda* or Samoa* or "Sao Tome*" or Principe*	3314
S66	Senegal* or "Sierra Leone*" or "Solomon Island*"	1770
S67	Somalia* or Sudan* or "Sri Lanka*"	3845
S68	Swaziland* or Syria* or Tajik*	3328
S69	Tanzania* or Timor* or Togo* or Tonga*	3835
S70	Tunisia* or Turk*	54 767
S71	Uganda* or Ukrain*	7063
S72	Uzbek* or Vanuatu* or Vietnam*	9721
S74	Yemen* or Zambia* or Zimbabwe*	3839
S75	S12 OR S13 OR S14 OR S15 OR S16 OR S17 OR S18 OR S19 OR S20 OR S21 OR S22 OR S23 OR S24 OR S25 OR S26 OR S27 OR S28 OR S29 OR S30 OR S31 OR S32 OR S33 OR S34 OR S35 OR S36 OR S37 OR S38 OR S39 OR S40 OR S41 OR S42 OR S43 OR S44 OR S45 OR S46 OR S47 OR S48 OR S49 OR S50 OR S51 OR S52 OR S53 OR S54 OR S55 OR S56 OR S57 OR S58 OR S59 OR S60 OR S61 OR S62 OR S63 OR S64 OR S65 OR S66 OR S67 OR S68 OR S69 OR S70 OR S71 OR S72 OR S73 OR S74	643 526
S76	DE "Family Intervention" OR DE "Strategic Family Therapy" OR DE "Early Intervention" OR DE "Family Relations"	59 137
S77	TI ( ( Family OR Families ) ) OR AB ( ( Family OR Families ) )	425 729
S78	TI ( ( mother OR mothers ) ) OR AB ( ( mother OR mothers ) )	134 635
S79	TI ( ( father OR fathers ) ) OR AB ( ( father OR fathers ) )	51 272
S80	TI ( ( Parent OR parents ) ) OR AB ( ( Parent OR parents ) )	251 604
S81	TI “Primary caregiver” OR AB “Primary caregiver”	1648
S82	TI “Primary care giver” OR AB “Primary care giver”	51
S83	TI "home based" OR AB "home based"	5952
S84	TI "at home" OR AB "at home"	128 992
S85	TI "in the home" OR AB "in the home"	128 670
S86	S76 OR S77 OR S78 OR S79 OR S80 OR S81 OR S82 OR S83 OR S84 OR S85	748 900
S87	DE "Reproductive Health Care" OR DE "Family Planning" OR DE "Induced Abortion" OR DE "Prenatal Care" OR DE "Reproductive Health" OR DE "Reproductive Rights" OR DE "Abortion Laws" OR DE "Sexual Health" OR DE "Sexology" OR DE "Sexual Identity" OR DE "Sexual Violence" OR DE "Dating Violence" OR DE "Intimate Partner Violence" OR DE "Sex Offenses" OR DE "Sex Trafficking" OR DE "Sexual Harassment" OR DE "Sexual Development"	53 402

**Table:**

	**Query**	**Result**
S88	TI ( (condom or “safe sex” or condoms) ) OR AB ( (condom or “safe sex” or condoms) )	11 344
S89	TI ( (OC or Contraceptive) N2 pill OR (oral N2 contraceptive) ) OR AB ( (OC or Contraceptive) N2 pill OR (oral N2 contraceptive) )	1806
S90	TI ( ( abstain or abstinen* ) ) OR AB ( ( abstain or abstinen* ) )	22 659
S91	TI sex* N2 intercourse OR AB sex* N2 intercourse	5173
S92	TI ( pregnan* N2 (adolescen* or teen* or schoolchild*) ) OR AB ( pregnan* N2 (adolescen* or teen* or schoolchild*) )	4383
S93	TI ( pregnan* N3 (prevent* or interrupt* or unplanned or unwanted or mistimed) ) OR AB ( pregnan* N3 (prevent* or interrupt* or unplanned or unwanted or mistimed) )	4202
S94	TI ( (sexual* W0 transmi* or STI or STIs or STD or STDs or venereal ) ) OR AB ( (sexual* W0 transmi* or STI or STIs or STD or STDs or venereal ) )	12 596
S95	TI ( ( hiv or hiv‐1* or hiv‐2* or hiv1 or hiv2 or "human immunodeficiency virus" or "human immunedeficiency virus" or "human immuno‐deficiency virus" or "human immune‐deficiency virus" or ((human W0 immun*) AND (“deficiency virus")) or "acquired immunodeficiency syndrome" or "acquired immunedeficiency syndrome" or "acquired immuno‐deficiency syndrome" or "acquired immune‐deficiency syndrome" or ((acquired W0 immun*) AND (“deficiency syndrome") ) OR AB ( ( hiv or hiv‐1* or hiv‐2* or hiv1 or hiv2 or "human immunodeficiency virus" or "human immunedeficiency virus" or "human immuno‐deficiency virus" or "human immune‐deficiency virus" or ((human W0 immun*) AND (“deficiency virus")) or "acquired immunodeficiency syndrome" or "acquired immunedeficiency syndrome" or "acquired immuno‐deficiency syndrome" or "acquired immune‐deficiency syndrome" or ((acquired W0 immun*) AND (“deficiency syndrome") )	61 732
S96	TI ( ( antiretroviral* or anti‐retroviral* or ARV*) N2 (complian* or adheren*) ) OR AB ( ( antiretroviral* or anti‐retroviral* or ARV*) N2 (complian* or adheren*) )	1774
S97	TI ( (hpv or papilloma virus* or papillomavirus*) N2 (vaccinat* or revaccinat* or immuniz* or immunis* or immunother* or inoculat* or innoculat* or prophyla*) ) OR AB ( (hpv or papilloma virus* or papillomavirus*) N2 (vaccinat* or revaccinat* or immuniz* or immunis* or immunother* or inoculat* or innoculat* or prophyla*) )	1193
S98	TI ( (sexual or spous* or “intimate partner”) N3 (violen* or abus* or rape) ) OR AB ( (sexual or spous* or “intimate partner”) N3 (violen* or abus* or rape) )	47 153
S99	TI ( ( pubert* or pubescen* ) ) OR AB ( ( pubert* or pubescen* ) )	9063
S100	TI ( menstruat* or menstrual* ) OR AB ( menstruat* or menstrual* )	7412
S101	TI ( abort* or miscarr* or (pregnan* N2 terminat*) ) OR AB ( abort* or miscarr* or (pregnan* N2 terminat*) )	9140
S102	TI ( sex* N2 (protected or unprotected or safe or unsafe or risk* or behavio*) ) OR AB ( sex* N2 (protected or unprotected or safe or unsafe or risk* or behavio*) )	47 684
S103	TI ( reproductive N2 (health or care or service*) ) OR AB ( reproductive N2 (health or care or service*) )	4986
S104	S87 OR S88 OR S89 OR S90 OR S91 OR S92 OR S93 OR S94 OR S95 OR S96 OR S97 OR S98 OR S99 OR S100 OR S101 OR S102 OR S103	222 083
S105	DE "Clinical Trials" OR DE "Randomized Clinical Trials" OR DE "Randomized Controlled Trials"	13 732
S106	TI non-randomized control trial OR AB non-randomized control trial	31
S107	TI natural N2 experiment* OR AB natural N2 experiment*	2534
S108	TI Quasi N2 experiment* OR AB Quasi N2 experiment*	16 485
S109	TI Quasiexperiment* OR AB Quasiexperiment*	474
S110	TI interven* N2 stud* OR AB interven* N2 stud*	32 727
S111	TI interven* N2 effect* OR AB interven* N2 effect*	50 430
S112	TI ( Clinical W0 (trial* OR stud*) ) OR AB ( Clinical W0 (trial* OR stud*) )	50 606
S113	TI Comparative W0 stud* OR AB Comparative W0 stud*	16 583
S114	TI ( (multicenter* OR multicentre*) N2 (stud* OR trial*) ) OR AB ( (multicenter* OR multicentre*) N2 (stud* OR trial*) )	7593
S115	TI ( Controlled N2 (stud* OR trial*) ) OR AB ( Controlled N2 (stud* OR trial*) )	78 049
S116	TI ( (randomised OR randomized) N2 (stud* OR trial*) ) OR AB ( (randomised OR randomized) N2 (stud* OR trial*) )	77 398
S117	TI ( RCT OR nRCT ) OR AB ( RCT OR nRCT )	11 017
S118	S105 OR S106 OR S107 OR S108 OR S109 OR S110 OR S111 OR S112 OR S113 OR S114 OR S115 OR S116 OR S117	239 560
S119	S11 AND S75 AND S86 AND S104 AND S118	589

**Table:** CINAHL PLUS (EBSCO) Search

	**Query**	**Results**
S1	(MH "Adolescent Parents") OR (MH "Adolescent Mothers") OR (MH "Adolescent Health Services") OR (MH "Adolescent Fathers") OR (MH "Adolescent Behavior") OR (MH "Adolescent Health")	30 026
S2	(MH "Child") OR (MH "Child Abuse, Sexual")	532 999
S3	TI Adolescen* OR AB Adolescen*	172 064
S4	TI youth* OR AB youth*	64 785
S5	TI teen* OR AB teen*	21 423
S6	TI juvenile* OR AB juvenile*	12 481
S7	TI emerg* N2 adult* OR AB emerg* N2 adult*	4911
S8	TI early N2 adult* OR AB early N2 adult*	4887
S9	TI ( ( young N2 (adult* or person or people or woman or women or man or men) ) OR AB ( ( young N2 (adult* or person or people or woman or women or man or men) )	94 837
S10	TI ( Child* OR boy* OR girl* ) OR AB ( Child* OR boy* OR girl* )	645 996
S11	S1 OR S2 OR S3 OR S4 OR S5 OR S6 OR S7 OR S8 OR S9 OR S10	1 024 329
S12	(MH "Developing Countries") OR (MH "Low and Middle Income Countries")	23 313
S13	TI ( "middle income*" W0 (countr* or nation or nations or econom*) ) OR AB ( "middle income*" W0 (countr* or nation or nations or econom*) )	13 995
S14	TI ( "Low and middle income*" W0 (countr* or nation or nations or econom*) ) OR AB ( "Low and middle income*" W0 (countr* or nation or nations or econom*) )	10 986
S15	TI ( developing W0 (countr* or nation or nations or econom*) ) OR AB ( developing W0 (countr* or nation or nations or econom*) )	18 523
S16	TI ( ("under developed" or underdeveloped) W0 (countr* or nation or nations or econom*) ) OR AB ( ("under developed" or underdeveloped) W0 (countr* or nation or nations or econom*) )	265
S17	TI ( "3rd world" W0 (countr* or nation or nations or econom*) ) OR AB ( "3rd world" W0 (countr* or nation or nations or econom*) )	1
S18	TI ( "third world" W0 (countr* or nation or nations or econom*) ) OR AB ( "third world" W0 (countr* or nation or nations or econom*) )	202
S19	TI ( LAMI W0 (countr* or nation or nations or econom*) ) OR AB ( LAMI W0 (countr* or nation or nations or econom*) )	20
S20	TI ( LMIC W0 (countr* or nation or nations or econom*) ) OR AB ( LMIC W0 (countr* or nation or nations or econom*) )	46
S21	TI ( "poor income" W0 (countr* or nation or nations or econom*) ) OR AB ( "poor income" W0 (countr* or nation or nations or econom*) )	1
S22	TI ( "low* income" W0 (countr* or nation or nations or econom*) ) OR AB ( "low* income" W0 (countr* or nation or nations or econom*) )	4006
S23	TI ( "less* developed" W0 (countr* or nation or nations or econom*) ) OR AB ( "less* developed" W0 (countr* or nation or nations or econom*) )	369
S24	TI ( "least developed" W0 (countr* or nation or nations or econom*) ) OR AB ( "least developed" W0 (countr* or nation or nations or econom*) )	91
S25	(MH "Asia") OR (MH "Asia, Southern") OR (MH "Asia, Southeastern") OR (MH "Asia, Western") OR (MH "Asia, Central")	8897
S26	(MH "Africa")	9361
S27	(MH "West Indies")	2369
S28	(MH "America") OR (MH "South America") OR (MH "Latin America") OR (MH "Central America")	6931
S29	(MH "Europe, Eastern")	949
S30	TI ( (South or Southern) W0 Asia* ) OR AB ( (South or Southern) W0 Asia* )	4769
S31	TI ( ("South east*" or Southeast*) W0 Asia* ) OR AB ( ("South east*" or Southeast*) W0 Asia* )	4180
S32	TI West* W0 Asia* OR AB West* W0 Asia*	147
S33	TI ( (Middle or Far) W0 East* ) OR AB ( (Middle or Far) W0 East* )	5545
S34	TI Africa* OR AB Africa*	87 612
S35	TI ( ( Latin or Central or South) W0 America* ) OR AB ( ( Latin or Central or South) W0 America* )	11 228
S36	TI ( Caribbean* or "West Indies" ) OR AB ( Caribbean* or "West Indies" )	5268
S37	TI East* W0 Europe* OR AB East* W0 Europe*	2444
S38	Afghan* or Angola* or Armenia*	6118
S39	Azerbaijan or Afghanistan or Algeria or Angola	6110
S40	Bangladesh* or Benin* or Bhutan*	9365

**Table:**

	**Query**	**Results**
S41	Bolivia* or "Burkina Faso*" or "Upper Volta*"	2401
S42	Burundi* or Brazil or Belarus or Bulgaria or Bhutan or Benin or Bosnia	65 560
S43	Botswana or Brazil OR Burkina	64 436
S44	"Cabo Verd*" or "Cape Verd*"	121
S45	Cambodia* or Cameroon* or "Central African Republic*	4419
S46	Chad* or Comoros or Comorian or Congo*	14 707
S47	"Cote d'Ivoire" or Colombia or China	95 882
S48	"Ivory Coast" or Ivorian*	145
S49	Djibouti* or “Democratic Republic of the Congo” or Dominica OR “Dominican Republic”	2716
S50	Ecuador	1636
S51	Egypt* or "El Salvador*"	6990
S52	Eritrea* or Ethiopia*	9320
S53	Gambia* or Georgia* or Ghana* or Guatemala*	8913
S54	Guinea* or Guyan* or Gabon or Guinea-Bissau	5462
S55	Haiti* or Hondura*	3742
S56	India* or Indonesia* or Iran or Iraq or Jordan*	150 464
S57	Kenya* or Kiribati* or Korea* or Kosov* or Kyrgyz*	9430
S58	Lesotho or Mosotho or Basotho	408
S59	Liberia* or Lao*	4410
S60	Madagasca* or Malagasy	622
S61	Malawi* or Mali or Mali's or Malian* or Mauritania* or Maldives	5531
S62	Micronesia* or Moldov* or Mongolia*	2132
S63	Morocc* or Mozambi* or Myanmar* or Burma* or Burmese*	6046
S64	Nepal* or Nicaragua* or Niger* or Nigeria or “North Marcedonia”	18 320
S65	Pakistan* or PNG* or Paraguay* or Philippines* or Filipino*	15 146
S66	Rwanda* or Samoa* or "Sao Tome*" or Principe*	3063
S67	Senegal* or "Sierra Leone*" or "Solomon Island*"	2683
S68	Somalia* or Sudan* or "Sri Lanka*"	7162
S69	Swaziland* or Syria* or Tajik*	3821
S70	Tanzania* or Timor* or Togo* or Tonga*	7359
S71	Tunisia* or Turk*	41 321
S72	Uganda* or Ukrain*	10 159
S73	Uzbek* or Vanuatu* or Vietnam*	8357
S74	"West Bank*" or Gaza* or Palestin*	2897
S75	Yemen* or Zambia* or Zimbabwe*	5985
S76	S12 OR S13 OR S14 OR S15 OR S16 OR S17 OR S18 OR S19 OR S20 OR S21 OR S22 OR S23 OR S24 OR S25 OR S26 OR S27 OR S28 OR S29 OR S30 OR S31 OR S32 OR S33 OR S34 OR S35 OR S36 OR S37 OR S38 OR S39 OR S40 OR S41 OR S42 OR S43 OR S44 OR S45 OR S46 OR S47 OR S48 OR S49 OR S50 OR S51 OR S52 OR S53 OR S54 OR S55 OR S56 OR S57 OR S58 OR S59 OR S60 OR S61 OR S62 OR S63 OR S64 OR S65 OR S66 OR S67 OR S68 OR S69 OR S70 OR S71 OR S72 OR S73 OR S74 OR S75	620 509
S77	"Family intervention"	696
S78	TI ( Family OR Families ) OR AB ( Family OR Families )	299 487
S79	TI ( mother OR mothers ) OR AB ( mother OR mothers )	99 561
S80	TI ( father OR fathers ) OR AB ( father OR fathers )	19 898
S81	TI ( Parent OR parents ) OR AB ( Parent OR parents )	130 845
S82	TI “Primary caregiver” OR AB “Primary caregiver”	1171
S83	TI “Primary care giver” OR AB “Primary care giver”	51
S84	TI "home based" OR AB "home based"	9151
S85	TI "at home" OR AB "at home"	158 443
S86	TI "in the home" OR AB "in the home"	15 990
S87	S77 OR S78 OR S79 OR S80 OR S81 OR S82 OR S83 OR S84 OR S85 OR S86	584 441
S88	(MH "Reproductive Health") OR (MH "Sexual Health") OR (MH "Child Abuse, Sexual") OR (MH "Sexual Abuse")	33 899
S89	TI ( condom or “safe sex” or condoms ) OR AB ( condom or “safe sex” or condoms )	11 313
S90	TI ( (OC or Contraceptive) N2 pill OR (oral N2 contraceptive) ) OR AB ( (OC or Contraceptive) N2 pill OR (oral N2 contraceptive) )	6056

**Table:**

	**Query**	**Results**
S91	TI ( abstain or abstinen* ) OR AB ( abstain or abstinen* )	12 959
S92	TI sex* N2 intercourse OR AB sex* N2 intercourse	3970
S93	TI ( pregnan* N2 (adolescen* or teen* or schoolchild*) ) OR AB ( pregnan* N2 (adolescen* or teen* or schoolchild*) )	4858
S94	TI ( pregnan* N3 (prevent* or interrupt* or unplanned or unwanted or mistimed) ) OR AB ( pregnan* N3 (prevent* or interrupt* or unplanned or unwanted or mistimed) )	7486
S95	TI ( sexual* W0 transmi* or STI or STIs or STD or STDs or venereal ) OR AB ( sexual* W0 transmi* or STI or STIs or STD or STDs or venereal )	21 104
S96	TI ( hiv or hiv‐1* or hiv‐2* or hiv1 or hiv2 or "human immunodeficiency virus" or "human immunedeficiency virus" or "human immuno‐deficiency virus" or "human immune‐deficiency virus" or ((human W0 immun*) AND (“deficiency virus")) or "acquired immunodeficiency syndrome" or "acquired immunedeficiency syndrome" or "acquired immuno‐deficiency syndrome" or "acquired immune‐deficiency syndrome" or ((acquired W0 immun*) AND (“deficiency syndrome" ) OR AB ( hiv or hiv‐1* or hiv‐2* or hiv1 or hiv2 or "human immunodeficiency virus" or "human immunedeficiency virus" or "human immuno‐deficiency virus" or "human immune‐deficiency virus" or ((human W0 immun*) AND (“deficiency virus")) or "acquired immunodeficiency syndrome" or "acquired immunedeficiency syndrome" or "acquired immuno‐deficiency syndrome" or "acquired immune‐deficiency syndrome" or ((acquired W0 immun*) AND (“deficiency syndrome" )	122 257
S97	TI ( antiretroviral* or anti‐retroviral* or ARV*) N2 (complian* or adheren* ) OR AB ( antiretroviral* or anti‐retroviral* or ARV*) N2 (complian* or adheren* )	2208
S98	TI ( (hpv or papilloma virus* or papillomavirus*) N2 (vaccinat* or revaccinat* or immuniz* or immunis* or immunother* or inoculat* or innoculat* or prophyla*) ) OR AB ( (hpv or papilloma virus* or papillomavirus*) N2 (vaccinat* or revaccinat* or immuniz* or immunis* or immunother* or inoculat* or innoculat* or prophyla*) )	4099
S99	TI ( (sexual or spous* or “intimate partner”) N3 (violen* or abus* or rape) ) OR AB ( (sexual or spous* or “intimate partner”) N3 (violen* or abus* or rape) )	23 822
S100	TI ( pubert* or pubescen* ) OR AB ( pubert* or pubescen* )	7556
S101	TI ( menstruat* or menstrual* ) OR AB ( menstruat* or menstrual* )	12 740
S102	TI ( abort* or miscarr* or (pregnan* N2 terminat*) ) OR AB ( abort* or miscarr* or (pregnan* N2 terminat*) )	23 869
S103	TI ( sex* N2 (protected or unprotected or safe or unsafe or risk* or behavio*) ) OR AB ( sex* N2 (protected or unprotected or safe or unsafe or risk* or behavio*) )	25 292
S104	TI ( reproductive N2 (health or care or service*) ) OR AB ( reproductive N2 (health or care or service*) )	10 936
S105	S88 OR S89 OR S90 OR S91 OR S92 OR S93 OR S94 OR S95 OR S96 OR S97 OR S98 OR S99 OR S100 OR S101 OR S102 OR S103 OR S104	261 947
S106	(MH "Randomized Controlled Trials") OR (MH "Clinical Trials")	314 476
S107	(MH "Quasi-Experimental Studies+") OR (MH "Experimental Studies+")	430 128
S108	TI natural N2 experiment* OR AB natural N2 experiment*	1441
S109	TI Quasi N2 experiment* OR AB Quasi N2 experiment*	15 342
S110	TI Quasiexperiment* OR AB Quasiexperiment*	703
S111	TI interven* N2 stud* OR AB interven* N2 stud*	40 104
S112	TI interven* N2 effect* OR AB interven* N2 effect*	50 823
S113	TI ( Clinical W0 (trial* OR stud*) ) OR AB ( Clinical W0 (trial* OR stud*) )	170 933
S114	TI Comparative W0 stud* OR AB Comparative W0 stud*	19 157
S115	TI ( (multicenter* OR multicentre*) N2 (stud* OR trial*) ) OR AB ( (multicenter* OR multicentre*) N2 (stud* OR trial*) )	43 178
S116	TI ( Controlled N2 (stud* OR trial*) ) OR AB ( Controlled N2 (stud* OR trial*) )	199 046
S117	TI ( (randomised OR randomized) N2 (stud* OR trial*) ) OR AB ( (randomised OR randomized) N2 (stud* OR trial*) )	251 468
S118	TI ( RCT OR nRCT ) OR AB ( RCT OR nRCT )	30 038
S119	S106 OR S107 OR S108 OR S109 OR S110 OR S111 OR S112 OR S113 OR S114 OR S115 OR S116 OR S117 OR S118	737 251
S120	S11 AND S76 AND S87 AND S105 AND S119	1065

**Table:** EMBASE (Ovid) Search

**#**	**Query**	**Results from 25 October 2023**
1	exp adolescent/	1 786 089
2	juvenile/	55 794
3	child/ or boy/ or girl/	2 136 768
4	adult child/	2050
5	young adult/	520 111
6	adolescen*.ti,ab,kf.	478 957
7	(youth* or teen* or juvenile*).ti,ab,kf.	284 166
8	(emerg* adj3 adult*).ti,ab,kf.	13 206
9	(early adj3 adult*).ti,ab,kf.	20 612
10	(young adj3 (adult* or person or people or woman or women or man or men)).ti,ab,kf.	310 929
11	(Child* or boy* or girl*).ti,ab,kf.	2 321 796
12	(Child* or boy* or girl*).ti,ab,kf.	2 321 796
13	or/1-12	4 467 268
14	developing country/	101 119
15	(middle income$ adj (countr$ or nation or nations or econom$)).ti,ab,kw.	40 612
16	(developing adj (countr$ or nation or nations or econom$)).ti,ab,kw.	98 037
17	((under developed or underdeveloped) adj (countr$ or nation or nations or econom$)).ti,ab,kw.	2078
18	(3rd world adj (countr$ or nation or nations or econom$)).ti,ab,kw.	27
19	(third world adj (countr$ or nation or nations or econom$)).ti,ab,kw.	1558
20	(LAMI adj (countr$ or nation or nations or econom$)).ti,ab,kw.	59
21	(LMIC adj (countr$ or nation or nations or econom$)).ti,ab,kw.	199
22	(poor income adj (countr$ or nation or nations or econom$)).ti,ab,kw.	6
23	(low$ income adj (countr$ or nation or nations or econom$)).ti,ab,kw.	12 833
24	(less$ developed adj (countr$ or nation or nations or econom$)).ti,ab,kw.	1832
25	(least developed adj (countr$ or nation or nations or econom$)).ti,ab,kw.	387
26	asia/ or far east/ or middle east/ or south asia/	95 311
27	africa/ or "africa south of the sahara"/ or north africa/	79 889
28	Caribbean/ or Caribbean Islands/	8789
29	"south and central america"/ or central america/ or south america/	41 404
30	Eastern Europe/	7545
31	((South or Southern) adj Asia$).ti,ab,kw.	18 065
32	((South East or Southeast$) adj Asia$).ti,ab,kw.	27 526
33	West$ Asia$.ti,ab,kw.	1415
34	((Middle or Far) adj East$).ti,ab,kw.	29 436
35	Africa$.ti,ab,kw.	368 054
36	((Latin or Central or South) adj America$).ti,ab,kw.	70 012
37	(Caribbean$ or West Indies).ti,ab,kw.	24 442
38	East$ Europe$.ti,ab,kw.	12 772
39	(Afghan$ or Angola$ or Armenia$).mp.	18 261
40	(azerbaijan or algeria).mp.	10 357
41	(Bangladesh$ or Benin$ or Bhutan$).mp.	37 686
42	(Bolivia$ or Burkina or Upper Volta$).mp.	13 305
43	(Burundi$ or Brazil or belarus or bulgaria or bhutan or benin or bosnia or Cabo Verd$ or Cape Verd$).mp.	213 209
44	Botswana.mp.	4116
45	(Cambodia$ or Cameroon$ or Central African Republic$).mp.	21 238
46	(Chad$ or Comoros or Comorian or Congo$).mp.	43 420
47	(Colombia or china).mp.	538 181
48	(Cote d'Ivoire or Ivory Coast or Ivorian$).mp.	6276
49	(Djibouti$ or Dominica$ or Egypt$ or El Salvador$).mp.	56 509
50	(Eritrea$ or Ethiopia$ or Ecuador or Gambia$).mp.	55 172
51	(Georgia$ or Ghana$ or Guatemala$).mp.	42 905
52	(Guinea$ or Guyan* or Gabon or Haiti$ or Hondura$).mp.	153 903
53	(India$ or Indonesia$ or Iran or Iraq or Jordan$).mp.	545 084
54	(Kenya$ or Kiribati$ or Korea$).mp.	209 622

**Table:**

**#**	**Query**	**Results from 25 October 2023**
55	(Lesotho or Mosotho or Basotho).mp.	1297
56	(Liberia$ or Madagasca$ or Malagasy).mp.	10 675
57	(Kosov$ or Kyrgyz$ or Lao$).mp.	11 958
58	(Malawi$ or Mali or Mali's or Malian$ or Mauritania$ or Maldives).mp.	20 363
59	(Micronesia$ or Moldov$ or Mongolia$).mp.	17 229
60	(Morocc$ or Mozambi$ or Myanmar$ or Burma$ or Burmese$).mp.	33 125
61	(Nepal$ or Nicaragua$ or Niger$ or nigeria or "north macedonia").mp.	110 989
62	(Pakistan$ or PNG$ or paraguay$ or Philippines$ or Filipino$).mp.	80 450
63	(Rwanda$ or samoa* or Sao Tome$ or Principe$).mp.	11 161
64	(Senegal$ or Sierra Leone$ or Solomon Island$).mp.	17 341
65	(Somalia$ or Sudan$ or Sri Lanka$).mp.	31 734
66	(Swaziland$ or Syria$ or Tajik$).mp.	24 632
67	(Tanzania$ or Timor$ or Togo$ or tonga$).mp.	28 209
68	(Tunisia$ or Turk$ or Uganda$ or Ukrain$).mp.	185 609
69	(Uzbek$ or Vanuatu$ or Vietnam$).mp.	32 509
70	(West Bank$ or Gaza$ or Palestin$).mp.	6364
71	(Yemen$ or Zambia$ or Zimbabwe$).mp.	22 265
72	or/14-71	2 865 624
73	exp family/	608 020
74	exp family relation/	123 570
75	family support/	996
76	exp parent/	285 284
77	(family or families).ti,ab,kf.	1 453 052
78	(mother or mothers).ti,ab,kf.	334 113
79	(father or fathers).ti,ab,kf.	64 923
80	(parent or parents).ti,ab,kf.	422 567
81	(primary adj (caregiver or care giver)).ti,ab,kf.	2616
82	home based.ti,ab,kf.	20 915
83	at home.ti,ab,kf.	95 914
84	in the home.ti,ab,kf.	52 725
85	or/73-84	2 339 904
86	reproductive health/	23 882
87	exp sexuality/	320 723
88	sexual trauma/	398
89	exp sexual assault/	41 975
90	sexual crime/	13 442
91	(condom or "safe sex" or condoms).ti,ab,kf.	27 616
92	(((OC or Contraceptive) adj3 pill) or (oral adj3 contraceptive)).ti,ab,kf.	17 359
93	(abstain or abstinen$).ti,ab,kf.	42 621
94	(sex$ adj3 intercourse).ti,ab,kf.	15 862
95	(pregnan$ adj3 (adolescen$ or teen$ or schoolchild$)).ti,ab,kf.	10 077
96	(pregnan$ adj4 (prevent$ or interrupt$ or unplanned or unwanted or mistimed)).ti,ab,kf.	24 837
97	(sexual$ transmi$ or STI or STIs or STD or STDs or venereal).ti,ab,kf.	80 715
98	(hiv or hiv-1$ or hiv-2$ or hiv1 or hiv2 or human immunodeficiency virus or human immunedeficiency virus or human immuno-deficiency virus or human immune-deficiency virus or (human immun$ and deficiency virus) or acquired immunodeficiency syndrome or acquired immunedeficiency syndrome or acquired immuno-deficiency syndrome or acquired immune-deficiency syndrome or (acquired immun$ and deficiency syndrome)).ti,ab,kf.	499 770
99	((antiretroviral$ or anti-retroviral$ or ARV$) adj3 (complian$ or adheren$)).ti,ab,kf.	5229
100	((hpv or papilloma virus$ or papillomavirus$) adj3 (vaccinat$ or revaccinat$ or immuniz$ or immunis$ or immunother$ or inoculat$ or innoculat$ or prophyla$)).ti,ab,kf.	12 582
101	(((sexual or spous$ or intimate partner) adj4 (violen$ or abus$)) or rape).ti,ab,kf.	51 429
102	(pubert$ or pubescen$).ti,ab,kf.	63 368
103	(menstruat$ or menstrual$).ti,ab,kf.	74 310

**Table:**

**#**	**Query**	**Results from 25 October 2023**
104	(abort$ or miscarr$ or (pregnan$ adj3 terminat$)).ti,ab,kf.	141 971
105	(sex$ adj3 (protected or unprotected or safe or unsafe or risk$ or behavio$)).ti,ab,kf.	79 295
106	(reproductive adj3 (health or care or service$)).ti,ab,kf.	30 509
107	or/86-106	1 200 459
108	exp clinical trial/	1 847 894
109	exp controlled study/	10 128 103
110	(natural adj3 experiment$).ti,ab,kf.	8097
111	(quasi adj3 experiment$).ti,ab,kf.	26 638
112	quasiexperiment$.ti,ab,kf.	1183
113	(interven* adj3 (stud$ or effect$)).ti,ab,kf.	236 213
114	((clinical or controlled) adj3 (stud$ or trial$)).ti,ab,kf.	1 656 077
115	comparative stud*.ti,ab,kf.	148 376
116	((multicenter$ or multicentre$) adj3 (stud$ or trial$)).ti,ab,kf.	211 187
117	((randomised or randomized) adj3 (stud$ or trial$)).ti,ab,kf.	823 188
118	(RCT or nRCT).ti,ab,kf.	58 007
119	or/108-118	11 841 240
120	13 and 72 and 85 and 107 and 119	9645
121	limit 120 to embase	6901

**Table:** Web of Science Search

#1	((((((((((((((TS=(Adolescen*)) OR AB=(Adolescen*)) OR TS=(youth*)) OR AB=(youth*)) OR TS=(teen*)) OR AB=(teen*)) OR TS=(juvenile*)) OR AB=(juvenile*)) OR TS=(emerg* N2 adult* )) OR AB=(emerg* N2 adult* )) OR TS=(early N2 adult*)) OR AB=(early N2 adult*)) OR AB=(( young N2 (adult* or person or people or woman or women or man or men))) OR TS=(Child* OR boy* OR girl*)) OR AB=(Child* OR boy* OR girl*))	7 139 490
#2	((((((((((((((((((((((((((((((((((((((((((((((((((((((((((((((((((((((((((((((((((((((TS=( ( "middle income*" W0 (countr* or nation or nations or econom*))) OR AB=( ( "middle income*" W0 (countr* or nation or nations or econom*))) OR TS=("Low and middle income*" W0 (countr* or nation or nations or econom*)) OR AB=("Low and middle income*" W0 (countr* or nation or nations or econom*)) OR TS=(developing W0 (countr* or nation or nations or econom*))) OR AB=(developing W0 (countr* or nation or nations or econom*))) OR TS=(("under developed" or underdeveloped) W0 (countr* or nation or nations or econom*))) OR AB=(("under developed" or underdeveloped) W0 (countr* or nation or nations or econom*))) OR TS=("3rd world" W0 (countr* or nation or nations or econom*) )) OR AB=("3rd world" W0 (countr* or nation or nations or econom*) )) OR TS=("third world" W0 (countr* or nation or nations or econom*))) OR AB=("third world" W0 (countr* or nation or nations or econom*))) OR TS=(lamp W0 (countr* or nation or nations or econom*))) OR AB=(lamp W0 (countr* or nation or nations or econom*))) OR TS=(lsic W0 (countr* or nation or nations or econom*))) OR AB=(lsic W0 (countr* or nation or nations or econom*))) OR TS=("poor income" W0 (countr* or nation or nations or econom*))) OR AB=("poor income" W0 (countr* or nation or nations or econom*))) OR TS=("low* income" W0 (countr* or nation or nations or econom*))) OR AB=("low* income" W0 (countr* or nation or nations or econom*))) OR TS=("less* developed" W0 (countr* or nation or nations or econom*))) OR AB=("less* developed" W0 (countr* or nation or nations or econom*))) OR TS=("least developed" W0 (countr* or nation or nations or econom*))) OR AB=("least developed" W0 (countr* or nation or nations or econom*))) OR TS=((South or Southern) W0 Asia* )) OR AB=((South or Southern) W0 Asia* )) OR TS=(("South east*" or Southeast*) W0 Asia*)) OR AB=(("South east*" or Southeast*) W0 Asia*)) OR TS=(West* W0 Asia* )) OR AB=(West* W0 Asia* )) OR TS=((Middle or Far) W0 East*)) OR AB=((Middle or Far) W0 East*)) OR TS=(Africa*)) OR AB=(Africa*)) OR TS=(( Latin or Central or South) W0 America*)) OR AB=(( Latin or Central or South) W0 America*)) OR TS=(Caribbean* or "West Indies")) OR AB=(Caribbean* or "West Indies")) OR TS=(East* W0 Europe*)) OR AB=(East* W0 Europe*))))))))))))))))))))))))))))))))))))))))))))))))))))	1 741 464
#3	(((((((((((((((((((TS=(Afghan* or Angola* or Armenia*)) OR AB=(Afghan* or Angola* or Armenia*)) OR TS=(Azerbaijan or Afghanistan or algesia or Angola)) OR AB=(Azerbaijan or Afghanistan or algesia or Angola)) OR TS=(Bangladesh* or begin* or butan*)) OR AB=(Bangladesh* or begin* or butan*)) OR TS=(Bolivia* or “burking Faso*” or “Upper Volta*”)) OR AB=(Bolivia* or “burking Faso*” or “Upper Volta*”)) OR TS=(Burundi* or Brazil or Belarus or Bulgaria or butan or begin or boscia)) OR AB=(Burundi* or Brazil or Belarus or Bulgaria or butan or begin or boscia)) OR TS=(boisiana or Brazil OR burking)) OR AB=(boisiana or Brazil OR burking)) OR TS=(“Cabo Verd*” or “Cape Verd*”)) OR AB=(“Cabo Verd*” or “Cape Verd*”)) OR TS=(Cambodia* or Cameroon* or “Central African Republic*”)) OR AB=(Cambodia* or Cameroon* or “Central African Republic*”)) OR TS=(Chad* or comoris or cambrian or Congo*)) OR AB=(Chad* or comoris or cambrian or Congo*)) OR TS=(“Cote d'Ivoire" or Colombia or China”)) OR AB=(“Cote d'Ivoire” or Colombia or China)	2 932 532
#4	((((((((((((((((((((((((((TS=(“Ivory Coast” or Ivorian*)) OR AB=("Ivory Coast" or Ivorian*)) OR TS=(Djibouti* or “Democratic Republic of the Congo” or Dominica OR “Dominican Republic”)) OR AB=(Djibouti* or “Democratic Republic of the Congo” or Dominica OR “Dominican Republic”)) OR TS=(equator)) OR AB=(equator)) OR TS=(Egypt* or "El Salvador*")) OR AB=(Egypt* or "El Salvador*")) OR TS=(Eritrea* or Ethiopia*)) OR AB=(Eritrea* or Ethiopia*)) OR TS=(Gambia* or Georgia* or Ghana* or Guatemala*)) OR AB=(Gambia* or Georgia* or Ghana* or Guatemala*)) OR TS=(Guinea* or Guyan* or gabion or Guinea-Bissau)) OR AB=(Guinea* or Guyan* or gabion or Guinea-Bissau)) OR TS=(Haiti* or Hondura*)) OR AB=(Haiti* or Hondura*)) OR TS=(India* or Indonesia* or Iran or Iraq or Jordan*)) OR AB=(India* or Indonesia* or Iran or Iraq or Jordan*)) OR TS=(Kenya* or Kiribati* or Korea* or Kosov* or Kyrgyz*)) OR AB=(Kenya* or Kiribati* or Korea* or Kosov* or Kyrgyz*)) OR TS=(leastho or mesotho or basethe)) OR AB=(leastho or mesotho or basethe)) OR TS=(Liberia* or Lao*)) OR AB=(Liberia* or Lao*)) OR TS=(Madagasca* or malanase)) OR AB=(Madagasca* or malanase))	3 779 382

**Table:** Web of Science Search

#5	(((((((((((((((((((((((((((((TS=(Malawi* or Mali or Mali's or Malian* or Mauritania* or maladies)) OR AB=(Malawi* or Mali or Mali's or Malian* or Mauritania* or maladies)) OR TS=(Micronesia* or Moldov* or Mongolia*)) OR AB=(Micronesia* or Moldov* or Mongolia*)) OR TS=(Morocc* or Mozambi* or Myanmar* or Burma* or Burmese*)) OR AB=(Morocc* or Mozambi* or Myanmar* or Burma* or Burmese*)) OR TS=(Nepal* or Nicaragua* or Niger* or Nigeria or “North Marcedonia”)) OR AB=(Nepal* or Nicaragua* or Niger* or Nigeria or “North Marcedonia”)) OR TS=(Pakistan* or PNG* or Paraguay* or Philippines* or Filipino*)) OR AB=(Pakistan* or PNG* or Paraguay* or Philippines* or Filipino*)) OR TS=(Rwanda* or Samoa* or "Sao Tome*" or Principe*)) OR AB=(Rwanda* or Samoa* or "Sao Tome*" or Principe*)) OR TS=(Senegal* or "Sierra Leone*" or "Solomon Island*")) OR AB=(Senegal* or "Sierra Leone*" or "Solomon Island*")) OR TS=(Somalia* or Sudan* or "Sri Lanka*")) OR AB=(Somalia* or Sudan* or "Sri Lanka*")) OR TS=(Swaziland* or Syria* or Tajik*)) OR AB=(Swaziland* or Syria* or Tajik*)) OR TS=(Tanzania* or Timor* or Togo* or Tonga*)) OR AB=(Tanzania* or Timor* or Togo* or Tonga*)) OR TS=(Tunisia* or Turk*)) OR AB=(Tunisia* or Turk*)) OR TS=(Uganda* or Ukrain*)) OR AB=(Uganda* or Ukrain*)) OR TS=(Uzbek* or Vanuatu* or Vietnam*)) OR AB=(Uzbek* or Vanuatu* or Vietnam*)) OR TS=("West Bank*" or Gaza* or Palestin*)) OR AB=("West Bank*" or Gaza* or Palestin*)) OR TS=(Yemen* or Zambia* or Zimbabwe*)) OR AB=(Yemen* or Zambia* or Zimbabwe*)	1 929 887
#6	(((((((((((((((((TS=(Family OR Families)) OR AB=(Family OR Families)) OR TS=(mother OR mothers)) OR AB=(mother OR mothers)) OR TS=(father OR fathers )) OR AB=(father OR fathers )) OR TS=(Parent OR parents)) OR AB=(Parent OR parents)) OR TS=(“Primary caregiver”)) OR AB=(“Primary caregiver”)) OR TS=(“Primary care giver”)) OR AB=(“Primary care giver”)) OR TS=("home based")) OR AB=("home based")) OR TS=("at home")) OR AB=("at home")) OR TS=("in the home")) OR AB=("in the home")	5 136 175
#7	(((((((((((((((TS=( condom or “safe sex” or condoms)) OR AB=( condom or “safe sex” or condoms)) OR TS=(( (OC or Contraceptive) N2 pill)) OR AB=(( (OC or Contraceptive) N2 pill)) OR TS=(abstain or abstinen*)) OR AB=(abstain or abstinen*)) OR TS=( sex* N2 intercourse )) OR AB=( sex* N2 intercourse )) OR TS=(pregnan* N2 (adolescen* or teen* or schoolchild*))) OR AB=(pregnan* N2 (adolescen* or teen* or schoolchild*))) OR TS=(pregnan* N3 (prevent* or interrupt* or unplanned or unwanted or mistimed))) OR AB=(pregnan* N3 (prevent* or interrupt* or unplanned or unwanted or mistimed))) OR TS=(sexual* W0 transmi* or STI or STIs or STD or STDs or venereal ))) OR AB=(sexual* W0 transmi* or STI or STIs or STD or STDs or venereal ))))	207 788
#8	(TS=((hiv OR hiv1* OR hiv2* OR hiv1 OR hiv2 OR "human immunodeficiency virus" OR "human immunedeficiency virus" OR "human immuno‐deficiency virus" OR "human immune‐deficiency virus" OR ((human NO immun*) AND ("deficiency virus")) OR "acquired immunodeficiency syndrome" OR "acquired immunedeficiency syndrome" OR "acquired immuno‐deficiency syndrome" OR "acquired immune‐deficiency syndrome" OR ((acquired NO immun*) AND ("deficiency syndrome")))) OR (AB=((hiv OR hiv1* OR hiv2* OR hiv1 OR hiv2 OR "human immunodeficiency virus" OR "human immunedeficiency virus" OR "human immuno‐deficiency virus" OR "human immune‐deficiency virus" OR ((human NO immun*) AND ("deficiency virus")) OR "acquired immunodeficiency syndrome" OR "acquired immunedeficiency syndrome" OR "acquired immuno‐deficiency syndrome" OR "acquired immune‐deficiency syndrome" OR ((acquired NO immun*) AND ("deficiency syndrome"))))))	851 081
#9	(((((((((((((((TS=(( antiretroviral* or anti‐retroviral* or ARV*) N2 (complian* or adheren*) )) OR AB=(( antiretroviral* or anti‐retroviral* or ARV*) N2 (complian* or adheren*) )) OR TS=((hpv or papilloma virus* or papillomavirus*) N2 (vaccinat* or revaccinat* or immuniz* or immunis* or immunother* or inoculat* or innoculat* or prophyla*))) OR AB=((hpv or papilloma virus* or papillomavirus*) N2 (vaccinat* or revaccinat* or immuniz* or immunis* or immunother* or inoculat* or innoculat* or prophyla*))) OR TS=((sexual or spous* or “intimate partner”) N3 (violen* or abus* or rape))) OR AB=((sexual or spous* or “intimate partner”) N3 (violen* or abus* or rape))) OR TS=(pubert* or pubescen*)) OR AB=(pubert* or pubescen*)) OR TS=(menstruat* or menstrual* )) OR AB=(menstruat* or menstrual* )) OR TS=(abort* or miscarr* or (pregnan* N2 terminat*))) OR AB=(abort* or miscarr* or (pregnan* N2 terminat*))) OR TS=(sex* N2 (protected or unprotected or safe or unsafe or risk* or behavio*))) OR AB=(sex* N2 (protected or unprotected or safe or unsafe or risk* or behavio*))) OR TS=(reproductive N2 (health or care or service*))) OR AB=(reproductive N2 (health or care or service*))	484 110

**Table:** Web of Science Search

#10	(((((((((((((((((((((TS=(natural N2 experiment*)) OR TS=(natural N2 experiment*)) OR TS=(Quasi N2 experiment*)) OR AB=(Quasi N2 experiment*)) OR TS=(Quasiexperiment*)) OR AB=(Quasiexperiment*)) OR TS=(interven* N2 stud*)) OR AB=(interven* N2 stud*)) OR TS=(interven* N2 effect*)) OR TS=(interven* N2 effect*)) OR TS=(Clinical W0 (trial* OR stud*))) OR AB=(Clinical W0 (trial* OR stud*))) OR TS=(Comparative W0 stud*)) OR AB=(Comparative W0 stud*)) OR TS=((multicenter* OR multicentre*) N2 (stud* OR trial*))) OR AB=((multicenter* OR multicentre*) N2 (stud* OR trial*))) OR TS=(Controlled N2 (stud* OR trial*))) OR AB=(Controlled N2 (stud* OR trial*))) OR TS=((randomised OR randomized) N2 (stud* OR trial*))) OR AB=((randomised OR randomized) N2 (stud* OR trial*))) OR TS=(RCT OR nRCT)) OR AB=(RCT OR nRCT)	71 482
	#2 OR #3 OR #4 OR #5	8 842 468
	**#7 OR #8 OR #9**	1 466 851
	**#1 AND #6 AND #10 AND #11 AND #12**	**63**
